# Comparative genomics of blood and faecal *E. coli* and *K. pneumoniae* isolates from neonates with bloodstream infections in Tanzania

**DOI:** 10.1038/s42003-025-09008-5

**Published:** 2025-11-18

**Authors:** Richard N. Goodman, Sabrina J. Moyo, Ilinca Memelis, Aakash Khanijau, Joel Manyahi, Upendo O. Kibwana, Said Aboud, Bjørn Blomberg, Nina Langeland, Adam P. Roberts

**Affiliations:** 1 Department of Tropical Disease Biology, Liverpool School of Tropical Medicine, Liverpool, UK; 2 Department of Clinical Science, University of Bergen, Bergen, Norway; 3 Department of Microbiology and Immunology, Muhimbili University of Health and Allied Sciences, Dar es Salaam, Tanzania; 4 National Institute of Medical Research (NIMR), Dar es Salaam, Tanzania; 5 Department of Medicine, Haukeland University Hospital, Bergen, Norway; 6 Norwegian Institute of Public Health, Oslo, Norway

**Keywords:** Comparative genomics, Bacterial genetics

## Abstract

Bloodstream infections (BSIs) are a major cause of hospitalisation and death for children under the age of five in sub-Saharan Africa, with Gram-negative bacteria such as *Klebsiella pneumoniae* and *Escherichia coli* among the most common causative agents. These bacteria usually colonise the human gastrointestinal (GI) tract, which has been identified as a reservoir for invasive infections into extra-intestinal environments such as the urinary tract and bloodstream. In this study we used comparative genomics to compare hybrid genome assemblies of blood and faecal bacterial isolates taken from the same patients (all neonates under 19 days old) to determine if the BSI associated isolates and the GI tract associated isolates were related. Multiple pairs of highly related *E. coli* and *K. pneumoniae* were found, suggesting that translocation between the GI tract and the bloodstream occurred in multiple cases of BSI. We also highlight key virulence genes and acquired mutations that are indicative of pathogenic strains capable of BSI. These findings expand our understanding of the Gram-negative bacteria involved in BSI pathogenicity and could help guide targeted interventions to prevent future BSI infections in neonates.

## Abstract (web summary)

Genomic comparison of *E. coli* and *K. pneumoniae* isolated from faeces and blood of the same neonatal patients revealed highly related pairs, suggesting translocation between the gastrointestinal tract and the bloodstream occurred in multiple patients.

## Introduction

Bloodstream infections (BSI) are a major cause of sepsis, hospitalisation and death for children under the age of 5. Worldwide, there were an estimated 2.9 million sepsis-related deaths in 2017, with the highest burden in sub-Saharan Africa^1^. In this region, neonates can be particularly susceptible to sepsis caused by invasive bacterial infections due to co-morbidities such as HIV infection, malnutrition, malaria and sickle-cell disease^2–4^. It is often difficult to distinguish between febrile illness in infants caused by bacteria and malaria or other infectious agents within low-middle-income countries (LMICs), making them hard to diagnose and treat^5–7^. Gram-negative bacteria, including the Enterobacterales *Klebsiella pneumoniae* and *Escherichia coli*, were shown to be the main causative agents of neonatal sepsis in a study of several LMICs in Africa and South Asia^8^.

*K. pneumoniae* and *E. coli* both make up part of the human commensal, intestinal microbiota, and colonisation of the neonatal gastrointestinal (GI) tract with such organisms occurs rapidly after birth, influenced by the maternal flora and peri-partum environment. In the context of neonatal sepsis, the colonised GI tract has been identified as a reservoir for BSI^9^. This can arise from direct bacterial translocation from the gastrointestinal (GI) tract to the blood across the gut epithelium, or via secondary colonisation and infection of other body sites such as the skin, respiratory or urinary tract^10,11^. Bacterial translocation through the intestinal mucosal barrier into the bloodstream can occur during gut dysbiosis^12^ or increased intestinal permeability^13^. Premature neonates and those born with low birth weight are particularly vulnerable, as immature immune and gastrointestinal systems can lead to breach of the mucosal barrier^11,14,15^, and antibiotic exposure seen in healthcare environments such as the neonatal intensive care unit can contribute to gut dysbiosis^16^. Once the intestinal mucosal barrier has been traversed and bacteria are in the blood, this can lead to sepsis. To survive within two distinct environments as diverse as the intestine and blood, *K. pneumoniae* and *E. coli* isolates may already contain all the genes they need, or they may require adaptive mutations or the acquisition of genes via mobile genetic elements^17^.

A previous study investigated the trends of bacteraemia amongst 2226 children under 5 years of age who were hospitalised with fever in Dar es Salaam, Tanzania^7^. This study builds on that work by directly comparing bacterial isolates concurrently taken from blood and faecal samples of individual neonates on the same day, to assess whether the faecal and blood isolates are likely related. Highly related *E. coli* and *K. pneumoniae* isolates would likely have translocated between body sites, either from the GI tract to the blood or from the blood to the GI tract.

Studies from the USA and Taiwan have analysed faecal metagenomes of pre-term and very low birth weight neonates, showing bacterial translocation in patients^11,12,18^. There have also been studies that used comparative genomics to compare *E. coli* and *K. pneumoniae* from the urinary tract and gut in patients in the USA^19,20^. There have been further studies exploring the virulence factors that contribute to bacterial translocation to extra-intestinal sites and subsequent infections in the urinary tract of patients in France^21^. However, to our knowledge, this is the first study that has directly compared hybrid assembled genomes of blood and faecal *E. coli* and *K. pneumoniae* isolates from the same patients at the genomic and molecular level and highlighted virulence genes and acquired mutations that may contribute to the transition between body sites within individual patient isolates. This is also the first study to investigate bacterial translocation between the GI tract and bloodstream in a sub-Saharan Africa setting. The hybrid assemblies provide unique structural and contextual information about genes and replicons, allowing an in-depth comparative genomics approach. These findings expand our understanding of the Gram-negative organisms causing BSI infections and the dynamic genetic landscape likely involved in the translocation between body sites.

## Results

### Typing of *E. coli* and *K. pneumoniae* isolated from blood and faeces

In a previous study, 2226 blood cultures were analysed from children below the age of 5 years hospitalised with fever in Dar es Salaam, Tanzania^7^. Two hundred of these children (9%) were enrolled in a parallel study where faecal samples were taken at the same time as the blood samples^22^. 40 of the 200 patients (20%) had both ESBL-positive faecal and blood isolates. Sixteen out of these 40 blood-faecal pairs of ESBL isolates (40%) displayed identical phenotypic antimicrobial susceptibility testing (AST) profiles^22^.

In this study, we analysed 13 of these 16 blood–faecal pairs from the same patients with identical AST profiles (one or more of the 3 remaining pairs could not be located)^22^. These blood and faecal isolates were sequenced and hybrid assembled (see Supplementary Table 1) to determine their relatedness and the likelihood of bacterial translocation from the gastrointestinal (GI) tract to the bloodstream, or vice versa (see Table 1). Therefore, in total 26 isolates were assessed as 13 faecal (FC) and blood (BL) pairs; 4 pairs were confirmed as *Escherichia coli* (*n* = 8), 8 pairs of *Klebsiella pneumoniae* (*n* = 16), and 1 “pair” consisting of *Klebsiella pneumoniae* and *Klebsiella quasipneumoniae*^23^ (*n* = 2). These were from neonatal patients under the age of 19 days with fevers lasting 1–3 days (Supplementary Table 2). When referencing the genomic data, the prefix FS refers to patient ID (e.g., FS2155), the prefix FSBL refers to the blood isolate (FSBL2155), and the prefix FSFC refers to the faecal isolate (FSFC2155).

**Table 1:** MLST profiles of the 13 paired blood and faecal isolates (*n* = 26)

	Patient ID	Faecal isolates								Highly related pair	Blood isolates							
		Isolate name	Species	Type (MLST)	Sublineage (cgMLST)	Clonal Group (cgMLST)	K-type	O-type	*wzi* allele		Isolate name	Species	Type (MLST)	Sublineage (cgMLST)	Clonal Group (cgMLST)	K-type	O-type	*Wzi* allele
*Klebsiella* spp.	FS0558	FSFC0558	*K. quasipneumoniae similipneumoniae*	^*^cf4f			–	3/3a	–	–	FSBL0558	*K. pneumoniae*	280	280	280	K23	O2afg	82
	FS1386	FSFC1386	*K. pneumoniae*	76	76	10,052	K10	O3/O3a	100	Y	FSBL1386	*K. pneumoniae*	76	76	10,052	K10	O3/O3a	100
	FS1448	FSFC1448	*K. pneumoniae*	48	48	48	K62	O1	–	Y	FSBL1448	*K. pneumoniae*	48	48	48	K62	O1	62
	FS1925	FSFC1925	*K. pneumoniae*	14	14	14	K2	O1	2	–	FSBL1925	*K. pneumoniae*	6104	11,353	12,373	–	O5	–
	FS2111	FSFC2111K	*K. pneumoniae*	985	29	985	K39	O1	39	–	FSBL2111	*K. pneumoniae*	39	39	39	K2	O1	2
	FS2112	FSFC2112	*K. pneumoniae*	39	39	39	K2	O1	2	Y	FSBL2112	*K. pneumoniae*	39	39	39	K2	O1	2
	FS2130	FSFC2130	*K. pneumoniae*	607	607	607	K25	O1	133	Y	FSBL2130	*K. pneumoniae*	607	607	607	K25	O1	133
	FS2155	FSFC2155	*K. pneumoniae*	429	323	429	K27	O4	187	–	FSBL2155	*K. pneumoniae*	3559	323	429	K27	O4	187
	FS2265	FSFC2665	*K. pneumoniae*	22	22	22	K9	O2afg	9	–	FSBL2265	*K. pneumoniae*	607	607	607	K25	O1	133

	Patient ID	Isolate name	Species	Type (MLST)	Phylogroup (Clermon typing)	Highly related pair	Isolate name	Species	Type (MLST)	Phylogroup (Clermon typing)
*E. coli*	FS1654	FSFC1654	*E. coli*	1193/*ce2a	B2	Y	FSBL1654	*E. coli*	1193/*ce2a	B2
	FS2071	FSFC2071	*E. coli*	131/506	B2	Y	FSBL2071	*E. coli*	131/506	B2
	FS2240	FSFC2240	*E. coli*	1193/53	B2	Y	FSBL2240	*E. coli*	1193/53	B2
	FS2258	FSFC2258	*E. coli*	1193/53	B2	Y	FSBL2258	*E. coli*	1193/53	B2

Multi-locus sequence typing (MLST) from Pasteur Institute, France, for *Klebsiella* species (https://bigsdb.pasteur.fr/klebsiella/) and Warwick University, UK, for *E. coli* (http://mlst.warwick.ac.uk/mlst/dbs/Ecoli). Core genome MLST (cgMLST) from Pasteur Institute, France, for *Klebsiella* species (https://bigsdb.pasteur.fr/klebsiella/cgmlst-lincodes/). O-types, K-types and wzi allele for *Klebsiella* species were predicted with Kaptive 64. For *E. coli* phylogroups were assigned using ClermonTyping 66. The highly related pairs indicate shared MLST and, for *Klebsiella* spp., cgMLST. FS2155 shared the same cgMLST but not MLST, so it is not defined as a highly related pair.

Firstly, the relatedness of the paired blood and faecal isolates was assessed using multi-locus sequence typing (MLST) and core genome MLST (cgMLST). Eight out of the thirteen pairs (4 *E. coli* and 4 *K. pneumoniae*) shared the same MLST, and nine shared the same cgMLST (4 *E. coli* and 5 *K. pneumoniae*). The discrepancy occurred with patient FS2155; the faecal (FSFC2155) and blood (FSBL2155) MLST profiles were ST426 and ST3559, respectively, but the sublineage (323) and clonal group (ST429) from cgMLST were the same for both, as well as the K-type, O-type and *wzi* capsular gene type (see Table 1). Of the four *K. pneumoniae* highly related pairs, all had the same within-pair K-type, O-type and *wzi* gene type. Of the four *E. coli* pairs, all isolates, both from blood and faeces, were of the phylogroup B2. The *E. coli* isolates from patient FS2071 belonged to ST131, and the *E. coli* isolates from patients FS1654, FS2246 and FS2258 belonged to ST1193.

### Determining genome similarity between paired isolates using average nucleotide identity (ANI) and core genome single-nucleotide polymorphisms (SNPs)

**Fig. 1:**
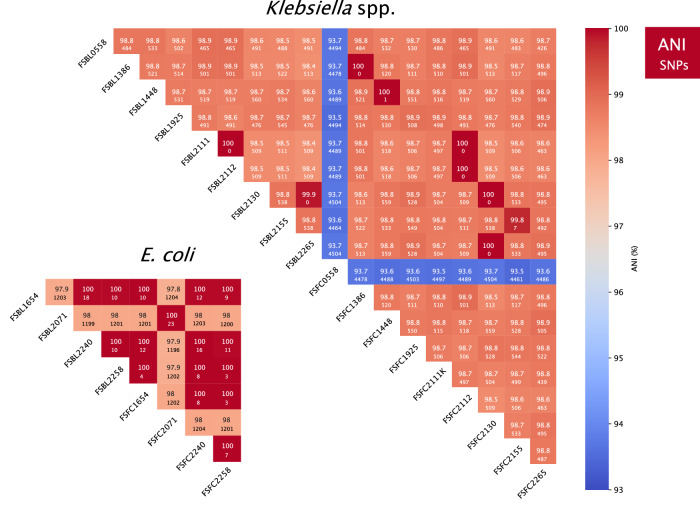
Average nucleotide identities (ANI) and core genome single-nucleotide polymorphisms (SNPs) across 4 *E. coli* and 9 *Klebsiella* spp. paired isolates. ANI was calculated using the BLAST alignment algorithm (ANIb). The core genome SNPs are displayed in each cell below the ANI %, this was calculated using snippy v4.6.3 and snp-dist using FSFC1386 as reference (see the “Methods” section). *Klebsiella* spp. are displayed together with 17 *Klebsiella pneumoniae* and 1 (FSFC0558) *Klebsiella quasipneumoniae* highlighted by the lines of blue.

To further assess the relatedness of the paired blood and faecal, average nucleotide identities (ANI) and core genome single nucleotide polymorphisms (SNP) distances were determined for *E. coli* and *Klebsiella* spp. (Fig. 1) and between all isolates (Supplementary Fig. 1). Of the 9 pairs sharing the same MLST, 8 had an ANI of 99.99% or above and 23 SNPs or less (i.e. ≥99.99% ANI and ≤23 SNPs), with FS2155 isolates having an ANI of 99.78% and 7 SNPs (see Fig. 1). The ANI analysis also revealed close relatedness between isolates which were not part of the same pair. For example, the *K. pneumoniae* isolate FSBL2111 shared an ANI of 100% and 0 SNPs with both FSFC2112 and FSBL2112, indicating they are all the same strain. The *K. pneumoniae* isolate FSBL2265 shares an ANI of 99.88% with FSBL2130 (0 SNPs) and 100% (0 SNPs) with FSFC2130, indicating they are likely the same sequence type or possibly the same strain. The *E. coli* isolates FSBL1654 and FSBL2240 share an ANI of 100% with FSFC2258 (see Fig. 1).

### Phylogenetics reveals the genomic relatedness across isolates

**Fig. 2:**
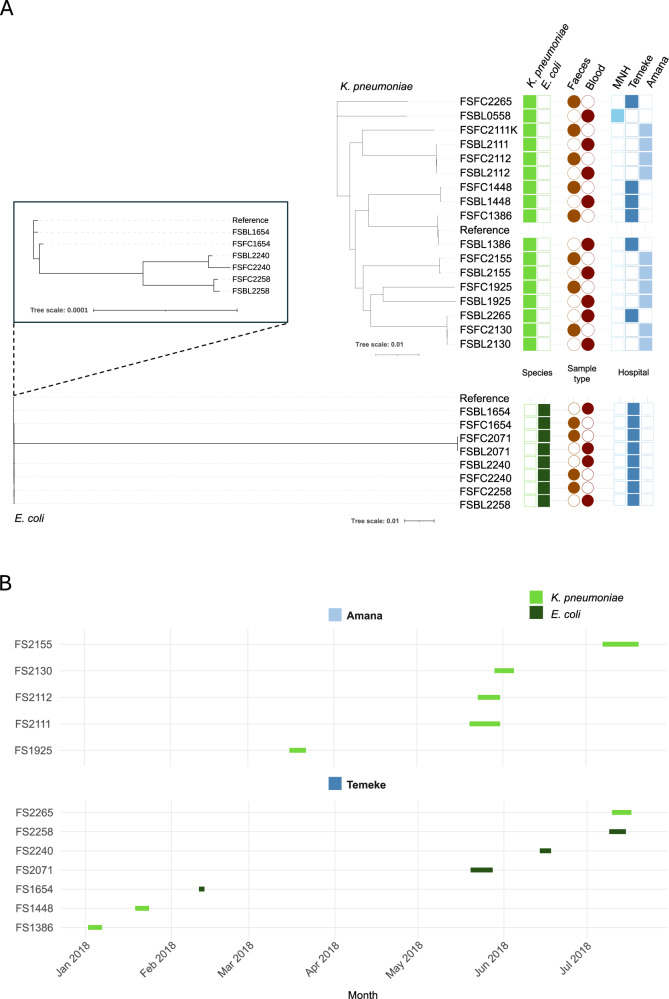
Core genome phylogenetic trees and admission timelines reveal relatedness across isolates. **A** Core genome phylogenetic tree of *K. pneumoniae* and *E. coli* isolates. The phylogenetic trees are based on SNPs derived from the core genome alignment of *K. pneumoniae* and *E. coli* isolates. The *Klebsiella pneumoniae* subsp. pneumoniae HS11286 genome (GCF_000240185.1) was used as a reference for *Klebsiella* *pneumoniae* isolates and the *Escherichia coli* str. K-12 substr. The MG1655 genome (GCF_000005845.2) was used as a reference for *E. coli* isolates. Branch lengths are representative of the number of SNPs between isolates. For the *E. coli* tree, FS2071 was removed to clearly visualise the relationship between the other paired isolates (shown in a black box). The coloured shapes indicate metadata such as species (green), sample type (brown/red) and hospital of origin (blue). MNH Muhimbili National Hospital. **B** Timelines from admission to discharge of neonatal patients in Temeke and Amana hospitals are represented by the start and end of the green lines in the timeline. MNH was excluded as only one patient was from this study site. The colours represent species consistent with the panel above.

Core genome trees were built based on SNP differences between the core genomes of closely related isolates (see Fig. 2A), all 8 pairs that share the same MLST, ≥99.99% ANI and ≤23 SNPs were on the same branch, with distances less than 0.00008 branch length between nodes. The isolates from FS2155 were also on the same branch. As with the ANI analysis, isolates from different patients were closely related; this included the *K. pneumoniae* isolate FSBL2111, which was on the same branch as FSBL2112 and FSFC2112, and the *K. pneumoniae* isolate FSBL2265, which was on the same branch as FSFC2130 and FSBL2130. Further to this, both blood and faecal *E. coli* isolates from FS1654, FS2240 and FS2258 were closely related on the tree.

Isolates from patients FS2111 and FS2112 were taken from the same hospital (Amana) within 3 days of each other (see Fig. 2B), indicating possible nosocomial transmission. Isolates from patient FS2265 came from Temeke hospital, and isolates from patient FS2130 came from Amana, so the similarity in sequence type is likely only incidental. Isolates from patients FS2258, FS1654 and FS2240 were all taken from Temeke hospital, but in different months of the year, so nosocomial transmission here would indicate an endemic strain within the hospital.

Subsequently, the eight pairs (4 *E. coli* and 4 *K. pneumoniae*) shown to have the same ST from MLST, an ANI ≥ 99.99% and ≤23 SNPs (i.e., isolates from FS1386, FS1448, FS1654, FS2071, FS2112, FS2130, FS2240 and FS2258) will be referred to as highly related pairs. Together, it is likely that each of these highly related pairs represents bacterial translocation of the same isolate either from the GI tract to the bloodstream or the bloodstream to the GI tract.

### Comparison of AMR, virulence and metal resistance genes between highly related blood and faecal paired isolates

We then looked to compare the antimicrobial resistance (AMR), virulence factor and metal resistance genes between the highly related pairs to determine whether any genes were acquired or lost in the translocation between body sites. The acquired AMR profiles did change in two highly related pairs (see Supplementary Data 1). The percentage identity to the database reference gene changed for *sul2* and *aph(3”)-lb* in FS1448 isolates and there was a gain of *dfrA17* on an integron in FSBL1654 compared to FSFC1654, the *dfrA17* gene confers resistance to trimethoprim (part of co-trimoxazole) (Supplementary Fig. 2). There were no differences in the genes conferring resistance to amoxycillin, gentamicin and ceftriaxone, which were the main antibiotics used to treat the patients (see Supplementary Table 2). All 8 highly related pairs shared the same virulence factors when compared against the VFDB database and metal resistance genes when compared against the MEGARES database (see Supplementary Data 1).

### Comparative genomics of SNPs between highly related blood and faecal paired isolates

**Fig. 3:**
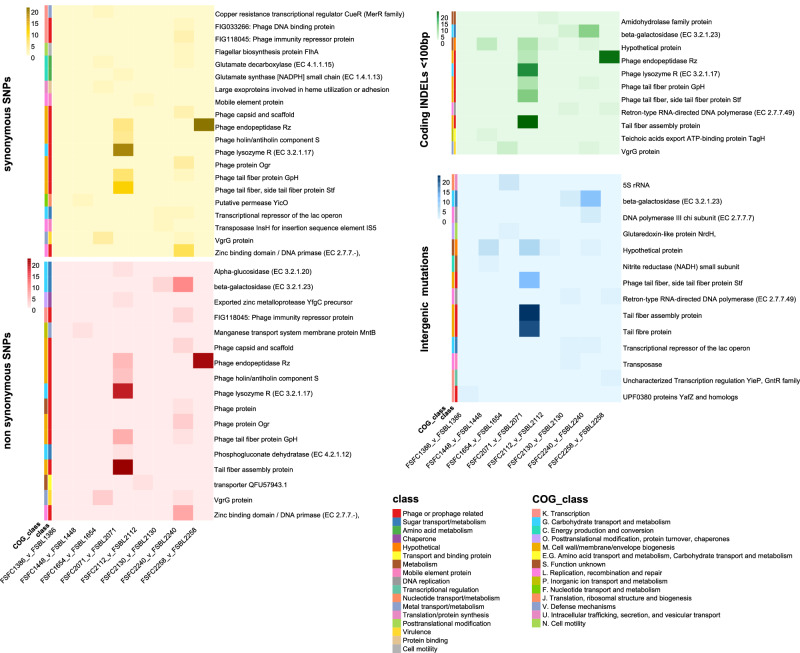
Predicted SNP mutations when comparing the blood genome against the faecal genome for a set of eight highly related paired isolates. All pairs compared with SNP mutations and INDELs <100 bp categorised by gene class, gene context and eggNOG clusters of orthologous genes (COG). SNPs and INDELs were identified with breseq. Heatmaps were visualised separately for synonymous SNPs, non-synonymous SNPs, INDELs <100 bp within the coding region, and intergenic mutations (both SNPs and INDELs). For intergenic mutations, the closest gene in bp to the SNP/INDEL is displayed.

We used breseq to run a genome comparison using the hybrid assembled faecal genome as a reference and the long reads of the blood isolates as a query for each of the highly related pairs, which had been confirmed to be highly related. This identified SNPs (both synonymous and non-synonymous), small INDELs (<100 bp) in the coding region and intergenic mutations which had been acquired in the blood isolate compared to the faecal isolate are shown in Fig. 3. The number of predicted mutations ranged from 1 to 516 across the nine pairs (Supplementary Table 3), with a median of 10, an average of 77.6 and standard deviation of 167. However, for some isolates, large numbers of SNPs were concentrated within a single coding DNA sequence (CDS), for example, in isolates from FS2071 there were 97 SNPs between the faecal and blood isolates with 19 in a gene encoding a phage lysozyme, 5 in a gene encoding a phage endopeptidase, and 16 in a gene encoding a phage tail fibre protein.

General trends across all the pairs can be seen in Fig. 3. SNPs were observed across pairs in genes involved in amino acid metabolism, DNA replication, metal transport/metabolism, sugar transport/metabolism and genes associated with mobile genetic elements and phages. Overall, most SNPs were found in genes related to prophages, which contributed to a range of functions, but mostly within genes encoding phage endopeptidase, phage lysozyme and genes involved in the assembly of the phage tail. Synonymous SNPs were found in the genes involved in the synthesis of the amino acid glutamate in FS2240 and FS1654. Mutations in and around genes of the *lac* operon were found in FS2240 and FS2130, which included non-synonymous SNPs, INDELs and intergenic mutations. Synonymous SNPs and INDELs were found in the virulence gene *vgrG* in both FS1654 and FS2240.

FS2155 is an outlier with 516 SNPs, and therefore, the SNPs are displayed separately in Supplementary Data 2. Non-synonymous SNPs of note were found in genes involved in sugar metabolism/transport, including genes of the *lac* operon (e.g., beta‑galactosidase) and those associated with galactose, glucose, lactose, sucrose and mannose. Genes encoding transcriptional regulators of the *lac* operon were also found to have non-synonymous SNPs. Ten non-synonymous SNPs and 3 intergenic mutations were found in the *fec* genes encoding for the iron dicitrate transport system, with 1 non-synonymous mutation found in the ferrichrome‑iron receptor gene. One of the non-synonymous SNPs in *fecA* was an A16T mutation, which is predicted not to be tolerated in the protein structure and therefore is likely to affect protein function. Together, this shows a complex picture of SNPs in a varied set of genes and gene classes contributing to, or as a result of, translocation between body sites.

### Insertions, deletions and mobile genetic element movement between highly related paired isolates

**Fig. 4:**
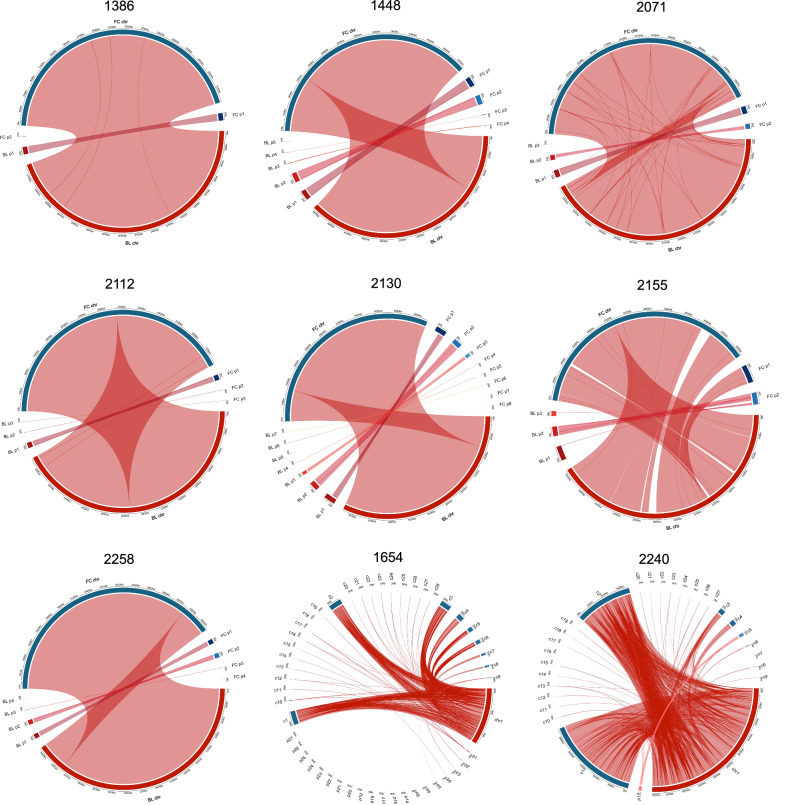
Whole genome comparison between the blood and faecal isolates for each highly related pair of isolates. Connections shown are homologous regions that share >99.9% sequence identity and are larger than 2 kb for all pairs except FS1654 and FS2240, where no size limit was imposed (i.e. >0 kb) due to the method of assembly. Each pair was aligned with BLASTn, blood isolate genome assemblies (red) were used as the query sequence and faecal isolate genome assemblies (blue) were used as the reference. Each genome is split into individual contigs, each with one chromosome and any extra-chromosomal contigs designated plasmids (even if a *rep* gene was absent). The exception is FS1654 and FS2240, where all contigs are numbered and not distinguished as a chromosome or plasmid. FC faecal isolates, BL blood isolate, chr chromosome, p plasmid, c contig.

We investigated any discrepancies across the entire genome between the highly related pairs, such as large insertions and deletions. The hybrid assemblies allowed us to compare contigs directly across pairs (see Supplementary Table 4) and a BLAST alignment of the blood and faecal isolates for each pair considered large insertions and deletions between and within contigs (Fig. 4). All the highly related pairs, except FS1654 and FS2140, have 1 chromosome and between 0 and 8 plasmids, however it is worth noting that the term “plasmid” here is used to refer to any extra-chromosomal contig, and not all extra-chromosomal contigs are likely to be plasmids, as some are <5 kb and contain no identifiable *rep* gene. Generally, across the pairs there is a loss or gain of phage genes and mobile genetic elements when translocating between body sites (see Supplementary Fig. 2). The blood and faecal genomes of FS2155 have the largest disparities of all paired samples in Fig. 4, with a 400 kb disparity in chromosome size however this could be explained by the insertion of FC p1 into the chromosome (see Supplementary Fig. 2). The connections in Fig. 4 show that all 8 highly related pairs (from patients FS1386, FS1448, FS1654, FS2071, FS2112, FS2130, FS2240 and FS2258) have chromosomes which share >99.9% sequence identity between blood and faecal isolates, providing further evidence that all these bloodstream isolates are clonally related to the isolates from the GI tract of their respective host.

### Hypermutator assay

We investigated whether FS2155 was a hypermutator strain, which could explain why there is a difference in the MLST, a lower ANI and large genomic changes between blood and faecal isolates. There was no clear difference between FS2155 and the other isolates in their ability to become resistant to rifampicin, indicating that the differences in the FS2155 isolates are not due to it being a hypermutator strain under the laboratory conditions under which it was tested. (Supplementary Table 5).

### Virulence, pathogenicity and invasiveness determinant genes across all isolates

**Fig. 5:**
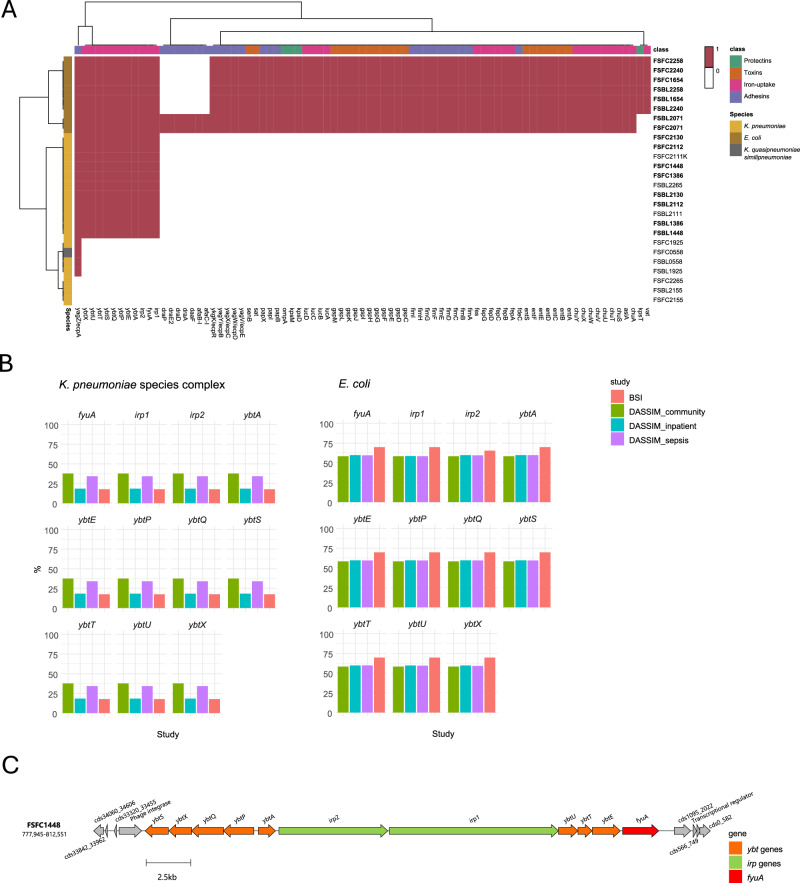
Virulence determinant genes in blood and faecal isolates across several datasets. **A** Paired and unpaired isolates from this study (*n* = 26) were screened against the virulence factor database, and the virulence genes present were visualised as a heatmap. Highly related pairs are in bold. The virulence genes were split into four classes. **B** The percentage of the *ybt*, *fyuA* and *ipA* genes in two datasets, BSI and DASSIM, from Blantyre, Malawi, containing 472 *E. coli* and 244 *K. pneumoniae* species complex isolates (KpSc). Out of the 472 *E. coli* 23, were BSI, 31 were DASSIM community, 334 were DASSIM sepsis and 84 were DASSIM inpatient. Out of the 244 KpSc 57, were BSI, 16 were DASSIM community, 138 were DASSIM sepsis and 33 were DASSIM inpatient. **C** The genomic structure of the *ybt* biosynthetic gene cluster in the FSFC1448 genome.

We screened all 26 isolates against the VirulenceFinder database to determine if there were certain genes that determined invasiveness/virulence in the translocation between body sites (Fig. 5A). The *E. coli* and *K. pneumoniae* isolates can be easily distinguished by the absence or presence of certain genes. With the *E. coli*, all 8 isolates have similar genes, which is expected as they are all part of phylogroup B2; the only difference is that FS2071 has 7 additional adhesion genes and lacks 2 genes, one involved in host defence evasion (*kpsT*) and another expressing a toxin (*vat*), which are found in the rest of the *E. coli*. With the *Klebsiella* spp., 61% of isolates (11/18) have the same set of virulence genes, these are all involved in iron-uptake, including a set of *ybt* genes, *irp* and *fyuA*. The *ybt* genes, involved in the production of the yersiniabactin siderophore, are present in the highly related paired isolates, which likely translocated between body sites, but are not present in the unpaired isolates. This is also the case for the *irp2*, encoding an iron regulatory protein, and *fyuA*, encoding the yersiniabactin receptor. This could indicate that these *ybt*, *fyuA* and *irp2* genes are important for the adaptation of *K. pneumoniae* in the bloodstream. These genes are always found on the chromosome of these isolates and are co-located within the same biosynthetic gene cluster (Fig. 5C). Of the rest of the *Klebsiella* isolates, 22% (4/18) have only one virulence gene, *yagZ*/*ecpA*, involved in adhesion, and 17% (3/18) had no detectable virulence gene from the virulence factor database.

To further investigate the association of the *ybt*, *fyuA* and *irp2* genes with BSI pathogenicity, we analysed two datasets, BSI and DASSIM, containing 716 *E. coli* and *K. pneumoniae* species complex isolates (KpSc) from Blantyre, Malawi. The BSI dataset contained blood isolates from sepsis patients and the DASSIM dataset contained faecal samples from community patients, inpatients and sepsis patients. We determined the percentage of these isolates containing the *ybt, fyuA* and *ipr2* genes (Fig. 5B) to see if they were overrepresented in bloodstream isolates compared to faecal isolates. Sixty-nine percent of *E. coli* isolates from the BSI dataset had all *ybt, fyuA* and *ipr2* genes, whereas in the DASSIM dataset, 58% were found in community patients, 59% found in inpatients and 59% found in sepsis patients. In this case, the *ybt, fyuA* and *ipr2* genes were overrepresented in the bloodstream infection isolates. With the *K. pneumoniae*, the picture is different, with the genes present in 18% of BSI isolates, and from the DASSIM faecal isolates, the genes are present in 18% of inpatients, 34% of sepsis patients and 38% of community patients.

## Discussion

In this study we used in-depth comparisons of hybrid assembled genomes with blood isolates and faecal samples taken from the same neonatal patients in Dar es Salaam, Tanzania^7,22^ to determine if the bacterial isolates translocated between body sites and what genomic changes, if any, accompanied this translocation event. We found that eight out of the 13 pairs (4 *E. coli* and 4 *K. pneumoniae*) shared the same MLST, had an ANI ≥ 99.99%, ≤23 SNPs and had a very small difference in branch length on the core genome phylogenetic tree. This high relatedness between the pairs indicates that the 4 *E. coli* and 4 *K. pneumoniae* isolates are likely clonal and either translocated from the GI tract to the blood, or vice versa, from the blood to the GI tract. The directionality cannot be deduced from our data since the faecal and blood samples were collected concurrently. In the context of neonatal sepsis, the colonised GI tract has previously been identified as a reservoir for BSI, and translocation of bacteria from the GI tract to the bloodstream has been reported from NICUs in the USA^9,11^. This is the most likely scenario for intestinal commensal organisms such as *E. coli* and *K. pneumoniae*. Translocation from the GI tract can either occur directly through the intestinal mucosal barrier into the bloodstream or via a secondary route by colonising other body sites such as the skin, respiratory or urinary tract^10,11^. The route of translocation (i.e., direct or secondary) cannot be deduced from our data; this would require extensive and invasive sampling of body sites in the same patients over time, something which was not feasible within our studies.

Alternatively, there are cases where bacteria have been shown to translocate from the bloodstream to the GI tract, such as in the case of *Salmonella enterica* serovar Typhi, a causative agent of Typhoid, which can infect the GI tract from the bloodstream via a secondary route of the gallbladder^24,25^. After initial invasion of the gut, *S*. Typhi invades the bloodstream either directly through the intestinal epithelial barrier^26^ or via the lymphatic system to cause systemic invasion^27^. *S*. Typhi can then invade the gallbladder directly from the bloodstream or by retrograde spread through the bile^28^. This can then lead to chronic colonisation of the gallbladder in a subset of individuals, where the *S*. Typhi is shed intermittently into the GI tract and in the faeces^26^. While a secondary route from the bloodstream to the GI tract is possible for *E. coli* and *K. pneumoniae* via organs such as the liver^29^, this route has been mostly reported with bacteria which are not commensals in the GI tract, such as *S*. Typhi, where it is a critical part of their life cycle. Further studies, including longitudinal cohort studies, should seek to determine the directionality and route of translocation and *K. pneumoniae* and *E. coli* in neonatal BSI infections in this setting.

The 4 *E. coli* highly-related pairs (from patients FS1654, FS2071, FS2240 and FS2258) were all of the phylogroup B2 (Table 1), which has been associated with pathogenic clones, particularly extraintestinal pathogenic *E. coli* (ExPEC)^10^ and has been indicative of urinary tract^21,30^ and bloodstream infection^31^. The 4 *K. pneumoniae* highly related pairs each shared the same within-pair K-type, O-type and *wzi* gene type, yet these differed between different pairs.

The blood and faecal *K. pneumoniae* isolates from patient FS2155 shared the same cgMLST, K-type, O-type and *wzi* gene type and 7 SNPs but had a different MLST and a lower ANI of 99.78% between blood and faecal isolates. Further comparison of whole genome sequences (Fig. 4) also indicated it is unlikely to be the same strain, as large parts of the chromosome did not share >99.9% sequence identity, which would be expected if the strain was the same and, in fact, with the eight highly related pairs, the entire chromosome shares 99.9% homology. Seemingly, the young age of the patient (1 day old) and the short time of fever (see Supplementary Table 2) would not accommodate enough generations of *K. pneumoniae* to allow for large number for SNPs between the FS2155 blood and faecal isolates, however no isolates were found to have an elevated mutation rate based on the results of the rifampicin mutation assay we carried out (Supplementary Table 5). Alternatively, we may have just isolated different but related isolates from the faeces and the blood, or the selection procedure may have missed the blood isolate in the faecal sample when in fact it was present.

The ANI/SNP comparisons and phylogenetic tree showed there was high relatedness between isolates from different patients in the same hospital, such as FS2111 and FS2112 from Amana and FS2258, FS1654 and FS2240 from Temeke (see Fig. 2), indicating potential nosocomial transmission between patients. Nosocomial transmission between patients within NICUs in the USA has been indicated prior to the onset of sepsis in another study^9^.

It is difficult to apply ANI thresholds to isolate relatedness, however a recent study showed >95% indicates the same species, >99.5% indicates the same sequence type and >99.99% indicates the same strain^32^. Our methodology also included SNP distances (see Fig. 1), which provides a clearer distinction between closely related isolates. In genomes containing millions of base pairs, percentages can provide misleading results. For example, the *E. coli* K-12 strain has a reference genome with a size of 4,639,221 bp, and 400 SNPs would still give an ANI of 99.99% but they may not be clonal. Nevertheless, there is no consensus on SNP thresholds to apply to bacterial relatedness. A recent study has shown that <100 SNPs represent isolate linkage across One Health sectors (i.e., animals, humans and the environment)^33^, while other studies have used lower thresholds to define relatedness in hospital settings, such as <25 SNPs per 5Mbp to indicate likely strain sharing of *K. pneumoniae*^34^ and <17 SNPs to infer *E. coli* transmission^35^. Therefore, in this study, we have chosen a multilayered approach to determine high relatedness between isolates. Going forward, we recommend the use of both ANI and SNP distances to determine isolate relatedness within a cohort of compared strains, as well as other methodologies presented in this study such as phylogenetics, variant calling and whole genome comparison.

The antibiotic resistance profiles only differed in two highly related pairs, with the gain of *dfrA17* in FSBL1654 compared to FSFC1654 being the only notable difference between all highly related pairs. Previously, it has been noted that the presence of ESBL-producing Gram-negative isolates in the faeces of hospitalised adults is associated with the subsequent onset of sepsis^9^ and all our blood isolates that caused bloodstream infections contained the common ESBL gene *bla*_CTX-M-15_, which confers resistance to 3rd generation cephalosporins and is the most common ESBL gene found in *E. coli* isolates worldwide^36^. This is likely due to the similarity in phenotypic antibiotic susceptibility profiles being a pre-requisite for selection of isolates in this study.

Comparative genomics showed there were common SNPs across patient-paired isolates in amino acid metabolism, DNA replication, metal transport/metabolism, sugar transport/metabolism and genes associated with mobile genetic elements and phages.

INDELs were found in genes of the *lac* operon in FS2240 and FS2130 (Fig. 3). INDEL mutations were also found in the *lac* operon and beta-galactosidase gene in FS2155, as well as genes involved in the metabolism of a wide set of sugars. *E. coli* can utilise a variety of sugars for growth in the GI tract^19^, and mutations in sugar metabolism/transport pathways have been shown to have an effect on the colonisation of the enterohemorrhagic *E. coli* strain EDL933 in the intestine of mice^37^. Since all the patients involved in this study were aged <19 days, the main sugar source for gut bacteria would be lactose from breast or formula milk; this could explain the mutations in the *lac* operon, as it would be redundant following a change of carbohydrate substrate.

When comparing highly related pairs, a synonymous mutation was found in a copper resistance transcriptional regulator in FS1654, and in FS2155, 10 non-synonymous mutations were found within the *fec* genes, which encode for the iron dicitrate transport system. This includes an A16T mutation in *fecA*, predicted to affect protein function. In a previous study, a separate non-synonymous mutation was found in *fecD* when assessing *E. coli* moving from the gut to the urinary tract^17^.

Most SNPs were found in genes related to phage/prophage (Fig. 3). This supports the whole genome comparative analysis in Fig. 4 and Supplementary Fig. 2, which show that differences in the genomes were mainly accounted for by movement of mobile elements, including phage. If the translocation occurred from the GI tract to the bloodstream, it could be the case that the movement from the nutrient-rich environment of the gut to the nutrient-sparse environment of the bloodstream causes a stress response in the cell, which in turn leads to widespread movement of mobile genetic elements (MGEs) and the activation of prophages and subsequent mobilisation of phages^38,39^. This activation of mobile genetic elements could be beneficial to bacteria, and the MGEs, allowing them to adapt to new environmental stresses by increasing genetic variability, as the movement of MGEs intracellularly can lead to gene knockouts or upregulation of mobilised genes when their genetic context changes^40^. Paradoxically, the activation of prophage to a phage would likely be deleterious for the bacterial host, and therefore, there could be a selection bottleneck for mutated phage genes in isolates successfully translocating between the gut and blood. In this case, the inactivation of genes involved in the replication of phages and subsequent lysis would be advantageous to the bacterium^41–43^.

This widespread movement of MGEs, including phage, could also enable the acquisition of beneficial traits from other bacteria; however, while there is a high abundance of plasmid transfer between bacteria in the gut^44^, there is unlikely to be high plasmid transfer in blood, as there is a high amount of transience. During conjugation, the cells must come into contact with one another and sustain the interaction long enough for genetic material to be exchanged^45^, and previously it has been shown that conjugation occurs at a higher rate in fixed conditions, such as biofilms, rather than liquid culture^46–48^.

We also looked to see if there were any genes in our blood isolates that were indicative of virulence, pathogenicity and invasiveness by screening all isolates against the virulence factor database (Fig. 5A). All *E. coli* isolates had the same virulence genes except the ones from FS2071, which had 7 additional adhesion genes and lacked 2 genes when compared to the others. The *Klebsiella* spp. had more variation in virulence genes, with 61%, including all the highly related pairs, having 11 genes involved in iron-uptake, and the rest (39%) only having 1 or 0 virulence genes. The additional 11 genes were variants of *ybt*, *irp* and *fyu* genes, which are all involved in iron uptake and metabolism. The *ybt* gene encodes for the yersiniabactin siderophore, which, unlike the enterobactin siderophore, can escape the host innate immune system protein sideocalin, and is associated with pathogenicity in *E. coli* and *K. pneumoniae*^49^. This could indicate that the yersiniabactin siderophore is key for the translocation of *K. pneumoniae* into the blood since all the highly related paired isolates had the yersiniabactin genes, and the unpaired isolates did not contain these genes. These unpaired isolates could unintentionally provide a background of faecal isolates that were not concurrently in the gut and the bloodstream and therefore show a potential difference in genes of intestinal *Klebsiella* isolates and extra-intestinal pathogenic *Klebsiella* isolates. However, since single colony picks do not capture the full strain diversity in the gut microbiota, we would need to use a more complete approach, such as metagenomics or whole plate sweeps, to assess the differences. We tested this further by including two datasets from Blantyre, Malawi (BSI and DASSIM), to compare the presence of the *ybt*, *iprA* and *fyuA* genes in 716 blood and faecal isolates and see if the genes were overrepresented in the bloodstream isolates. While there was no clear distinction between blood and faecal *Klebsiella* isolates, in *E. coli* isolates, there was a higher abundance of these genes in the BSI isolates when compared to faecal carriage isolates from DASSIM. Together, this indicates that important genes required for invasion into and/or persistence in the blood were already present in the gut isolates. This opens the possibility for diagnostic interventions, for example, certain patient groups, such as very low birthweight neonates, could have their stool screened by PCR for the presence of the *ybt*, *fyuA* and *irp2* genes to determine if they are predisposed to bloodstream infections. Since the same isolates were found in both blood and faecal samples, and the sampling of stool is much less invasive than blood sampling for neonates, this could make it easier for healthcare workers in limited resource settings to take stool samples to identify bacteria pre-disposed to cause BSI. This would require further investigation; however, if successful, this targeted intervention could contribute to the prevention of BSIs in neonates.

This study has several limitations. We selected our isolates using single colony picks; therefore, we may have missed some highly related paired isolates at the selection stage and were not able to capture strain diversity within the gut microbiota. The study was cross-sectional as the faecal and blood isolates were collected concurrently; therefore, the directionality of any bacterial translocation cannot be deduced from this data alone. A longitudinal study following children over time to establish any subsequent BSIs would have required a very large group of children due to the low incidence of BSI in an unselected population. Even though such a design is ideal, it is currently only theoretically feasible in the low-resource setting of this study. Not all the originally paired isolates could be found and sequenced; therefore, only 26 out of the original 32 isolates could be sequenced as pairs. By not sequencing these, we may have lost information about genes or SNPs involved in bacterial translocation to the blood, and we cannot predict how this may have biased the results. Further to this, the sample size of 13 paired samples from blood and faeces (*n* = 26) is small and as such we are not able to infer statistical significance from this low powered sample size. While most of the genomes were assembled with the same hybrid assembly method (hybracter), due to a lack of short read sequences two were assembled with flye and due to poor long read quality two were assembled with unicycler. The use of different genome assemblers for some isolates also introduces variability. While this did not affect sequence typing, ANI, SNP analysis or screening for the absence or presence of genes it did have an impact on the whole genome comparison between the blood and faecal isolates (Fig. 4).

To conclude, we have shown that 8 isolate pairs (4 *E. coli* and 4 *K. pneumoniae*) were highly related (same MLST, ≥99.99% ANI and ≤23 SNPs) and therefore translocated between body sites in neonatal patients with bloodstream infections. The changes that marked this transition varied between the isolate pairs but were mainly found in genes associated with mobile genetic elements and phages. It is possible that most of the genes required for persistence in the blood were also present in the highly related gut isolates, as a set of important iron uptake genes was shared across blood and faecal isolates within each species. This analysis also highlighted the potential involvement of the yersiniabactin siderophore in BSI caused by both *Klebsiella* spp. and *E. coli*. However, functional validation is required to confirm this. These findings expand our understanding of the Gram-negative bacteria causing BSI and provide potential genetic markers that could be utilised in targeted interventions to prevent future BSI infections in neonates.

## Methods

### Study design, sample collection and bacterial isolation

This hospital-based, prospective, cross-sectional study was conducted in Dar es Salaam, Tanzania, from March 2017 to July 2018. The study enroled children below 5 years of age who were admitted to hospital with fever (>37.5 °C) at three regional hospitals, Amana, Temeke, and Mwananyamala, and one tertiary hospital, Muhimbili National Hospital (MNH). The study analysed blood-cultures from 2226 children^7^, with 82% of participants under the age of two and about 10% having positive blood cultures. Sixty-two *K. pneumoniae* and 28 *E. coli* were isolated from the blood cultures of these patients, totalling 90 blood isolates. The collection and processing of blood samples and bacterial isolation have been described in detail previously^7^. Two hundred of these children (9%) were enroled in a parallel study, which also collected faecal samples^22^ at the same time as the blood samples using a systematic sampling approach, whereby after every 11 children, a child was selected. The faecal samples were collected as rectal swabs from each patient, and bacteria were isolated as previously described^22^. Among these, from the blood samples of the 200 patients, 83 children had confirmed Gram-negative Enterobacteriaceae BSI, and 117 children had negative blood cultures^22^. From the faecal samples, 46 *K. pneumoniae* and 64 *E. coli* were isolated from these 200 patients, totalling 110 faecal isolates. Fourty of the 200 patients (20%) had both ESBL-positive faecal and blood isolates. Sixteen out of these 40 blood–faecal pairs of ESBL isolates (40%) displayed identical phenotypic antimicrobial susceptibility testing (AST) profiles (same zone size in the disc diffusion method)^22^. We included 13 of these pairs with identical AST profiles in this study. Three pairs of the 16 were omitted as not all the isolates could be found and sequenced. The strains (*n* = 26) were sent to the Liverpool School of Tropical Medicine, UK and stored at −80 °C until the DNA was extracted and sequenced.

### Whole genome sequencing

Short-read sequencing was carried out by MicrobesNG (Birmingham, UK). Briefly, the DNA was extracted from the blood and faecal bacterial strains and prepared into DNA libraries using the Nextera XT Library Prep Kit (Illumina, CA, USA). The short-read sequencing was carried out using an Illumina machine (HiSeq X10 platform) according to the 250 bp paired-end protocol.

For long-read sequencing, DNA was extracted from blood isolates with the fire monkey weight high molecular weight (HMW) genomic (gDNA) DNA extraction kit (Revolugen, UK) and the faecal isolates with the Wizard HMW DNA extraction kit (Promega, WI, USA). Library prep was carried out using the SQK-NBD114.24 ligation sequencing and native barcoding kit according to the manufacturer's protocol (Oxford Nanopore Technologies, Oxford, UK). Sequencing was carried out using a FLO-MIN114 (R10.4.1) flow cell (Oxford Nanopore Technologies, Oxford, UK) on a MinION Mk1B sequencer, running for 72 h at a translocation speed of 400 bp/s. Data acquisition used the MinKNOW software (v22.08.9).

### Hybrid genome assembly

The raw data (fast5) was basecalled using guppy v6.4.2 with the super accuracy (sup) model specific to the flow cell (R10.4.1), motor protein (E8.2) and translocation speed (400 bp/s). Read quality was assessed with Nanoplot v1.38.1^50^. The basecalled files were demultiplexed with the guppy_barcoder from Guppy v6.3.8, specifying the SQK-NBD114-24 kit. The reads were assembled into de novo genome assemblies using either hybracter v0.6.0^51^, unicycler v0.4.8^52^ or flye v2.9.2^53^ (see Supplementary Table 1). The majority were assembled with hybracter (*n* = 22), FSFC1448 and FSBL1448 were assembled with flye as no short read match could be found, and FSFC1654 and FSFC2240 were assembled with unicycler due to the short reads being better quality than the long reads. Hybracter is an automated pipeline for long-read first hybrid assembly. This workflow runs initial quality control, assembles the genome from long reads with flye^53^ and plasmids with plassembler^54^, polishes with long reads using medaka (https://github.com/nanoporetech/medaka), polishes with short reads using polypolish^55^ and pypolca (https://github.com/gbouras13/pypolca) and returns an output of assembly files and statistics. This workflow has been shown to be more accurate than the existing gold-standard hybrid assembly software^51^. Assemblies were assessed for quality with seqkit^56^ (see Supplementary Table 1) and visualised with Bandage v0.8.1^57^.

### Genomic data analysis and phylogenetics

The assembled genomes were annotated using RAST^58^ and screened against the Resfinder v 2.4.0^59^, virulence factor database (VFDB)^60^ and MEGARes^61^ databases using abricate v1.0.1 (https://github.com/tseemann/abricate). Average nucleotide identities (ANI) between genomes were determined using JSpeciesWS v4.1.1^62^ and SNP distances were determined using snippy v4.6.3 and snp-dists v0.8.2 with the *K. pneumoniae* FSFC1386 isolate as reference for *Klebsiella* spp. isolates and the *E. coli* FSBL1654 isolate as a reference for the *E. coli* isolates. The heatmap showing both ANI and SNP distances between isolates was visualised with the pandas v2.2.3, matplotlib v3.9.4, seaborn v0.13.2 and numpy v2.2.0 packages in Python v3.10.12. Sequence typing used multilocus sequence types (MLST) and core genome MLST (cgMLST) schemes as part of the Pathogenwatch platform^63^. The MLST database from Pasteur Institute, France, was used for *Klebsiella* species (https://bigsdb.pasteur.fr/klebsiella/) and from Warwick University, UK, for *E. coli* (http://mlst.warwick.ac.uk/mlst/dbs/Ecoli). The cgMLST database from Pasteur Institute, France was used for *Klebsiella* species (https://bigsdb.pasteur.fr/klebsiella/cgmlst-lincodes/). O- and K-types for *Klebsiella* species were predicted with Kaptive^64^, along with *wzi* capsular gene type^65^. For *E. coli* phylogroups were assigned using ClermonTyping^66^, which is an in silico typing method based on the quadruplex PCR of the *arpA*, *chuA*, *yjaA* and *TspE4.C2* genes^67^.

A core genome phylogenetic tree of 17 *K. pneumoniae* and 8 *E. coli* isolates were built (*n* = 25), the *K. quasipneumoniae* isolate FSFC0558 was omitted from this analysis so that intra-species relationships could be analysed. The isolates were aligned to species-specific reference genomes using snippy v4.3.6, with the *Klebsiella pneumoniae* subsp. *pneumoniae* HS11286 genome (GCF_000240185.1) used as reference for *Klebsiella pneumoniae* isolates and the *Escherichia coli* str. K-12 substr. MG1655 genome (GCF_000005845.2) used as reference for *E. coli* isolates. The core SNP alignments were then combined using snippy-core. To retain only vertically inherited SNPs, Gubbins v2.3.4 was used to remove any recombinant regions from the alignment. A SNP-only alignment was extracted using snp-sites v2.5.1 with the -c option to select for A, C, G and T only. This was passed to IQ-tree v 1.6.1, which was used to construct a maximum-likelihood phylogenetic tree. The model was determined using the ModelFinder function of IQ-tree (-m MF), which calculated TVM + F + ASC + R2 as the best model for *K. pneumoniae* alignments and TVMe+ASC for *E. coli* alignments. The model was run using ultrafast 1000 bootstrap replicates (-bb 1000) and keeping identical data (-keep-ident). The phylogenetic tree and associated metadata were visualised using Interactive Tree Of Life (iTOL) v6^68^. The timelines were plotted from the admission metadata using dplyr v1.1.4 and ggplot v3.5.1.

Breseq v0.38.3^69^ was used to compare the RAST-annotated genomes from the faecal isolates against the long reads from the blood isolates, with the predicted mutations and unassigned missing coverage (MC) analysed from the output. The genes of interest were classified into clusters of orthologous genes (COGs)^70^ using eggNOG v5.0^71^, separated according to their mutations, and visualised with the pheatmap v1.0.12 package in R v4.3.1. Genes which were categorised as “Function unknown” within the COG framework were further investigated for putative functions, leading to an additional classification termed “class” inferred from gene annotations and BLAST^72^ searches against similar genes. We used SIFT^73^ to determine the predicted effect of genetic mutations on their protein function.

To determine the whole genome comparison between pairs, the hybrid assemblies from blood and faecal isolates were compared with BLASTn^72^, with the faecal isolate as query compared against a database made from the blood isolate genome. The BLAST output was wrangled with dplyr v1.1.4 to visualise as a circos diagram with the “genomic initialise” function of the circlize v0.4.16 package in R v4.3.1. Connections plotted were homologous regions that shared greater than 99.9% sequence identity and were larger than 2 kb, except for FS1654 and FS1654, where connections were homologous regions with > 99.9% sequence identity with no threshold limit on basepair size.

The 26 isolates from this study were screened against the virulence factor database (VFDB)^60^ using abricate v1.0.1 (https://github.com/tseemann/abricate). The output was plotted as a heatmap with the pheatmap v1.0.12 package in R v4.3.1. Two datasets, BSI and DASSIM, were analysed from Blantyre, Malawi, to determine the percentage of iron uptake virulence genes (*ybt*, *fyuA* and *ipA*) in blood and faecal isolates from another country in sub-Saharan Africa. Raw sequence read data were downloaded from the European Nucleotide Archive from the following projects: PRJEB8265, PRJEB28522, PRJEB26677, and PRJEB36486^74–76^. The BSI dataset contains isolates from blood, cerebral spinal fluid (CSF) and rectal swabs, collected as part of routine BSI surveillance; only the blood isolates were used for this analysis^74,75^. The DASSIM dataset involved stool sampling and ESBL-E isolate selection from patients with sepsis^76^. The sequence reads were quality controlled, trimmed and assembled as previously described^77^. 716 isolates (472 *E. coli* and 244 *Klebsiella* spp.) were screened against the VFDB^60^ using abricate v1.0.1. The data were separated by dataset and group (DASSIM_sepsis, DASSIM_inpatient, DASSIM_community, and BSI), and the percentage of occurrences was calculated by the number of gene occurrences divided by the total number in each group per species. Calculations were performed using the “summarise” function in dplyr v1.1.4, and the barplots were visualised with ggplot v3.5.1. The genomic structure of the *ybt* biosynthetic gene cluster from the RAST^58^ annotated FSFC1448 genome was visualised using clinker v0.0.31^78^.

### Calculation of mutation frequency

A rifampicin resistance assay was used to determine the mutation frequency for seven faecal isolates. The colonies were incubated at 37 °C in LB broth for 18 h, the cultures were normalised to an OD_600_ of 0.1, then incubated at 37 °C until the OD_600_ reached 0.6-0.8. The cultures were centrifuged at 4000 rpm to pellet the cells, then resuspended in 1 ml of PBS. From this resuspended solution, 100 ml was diluted in PBS to 10^−6^ and plated on LB agar plates, and 100 ml was plated on LB agar containing rifampicin (20 µg/ml). Plates were incubated at 37 °C for 16 h. CFU/ml was determined from colony counts of the total cell count plates. Mutation frequency was calculated by dividing the number of rifampicin-resistant colonies by the total number of viable cells plated (CFU/mL).

### Ethics declarations

The original sample collection was part of a study approved by the Senate Research and Publications Committee of Muhimbili University of Health and Allied Sciences, National Institute of Medical Research, Tanzania and the Regional Committee for Medical and Health Research Ethics (REK), Norway.

### Reporting summary

Further information on research design is available in the Nature Portfolio Reporting Summary linked to this article.

## Supplementary information

Supplementary Information

Description of Additional Supplementary Materials

Supplementary Data 1

Supplementary Data 2

Reporting Summary

## Data Availability

All data to reproduce this analysis are available through GitHub: https://rngoodman.github.io/blood-faecal-genomic-comparison and on a mirrored Zenodo repository: 10.5281/zenodo.17161336^79^. Reads from all isolates sequenced as part of this study have been submitted to the Sequence Read Archive (SRA) of the National Center for Biotechnology Information (NCBI) under the project ID PRJNA1254181. Supplementary Figs. 1, 2, Supplementary Tables 1–5 and Supplementary Data 1, 2 are accessible in the supplementary material.

## Code availability

All code to reproduce this analysis is publicly available through GitHub: https://rngoodman.github.io/blood-faecal-genomic-comparison and on a mirrored Zenodo repository: 10.5281/zenodo.17161336^79^.

## References

[1] Rudd, K. E. et al. Global, regional, and national sepsis incidence and mortality, 1990–2017: analysis for the Global Burden of Disease Study. *Lancet* **395**, 200–211 (2020). 31954465 10.1016/S0140-6736(19)32989-7 PMC6970225

[2] Scott, J. A. G. et al. Relation between falciparum malaria and bacteraemia in Kenyan children: a population-based, case-control study and a longitudinal study. *Lancet* **378**, 1316–1323 (2011). 21903251 10.1016/S0140-6736(11)60888-X PMC3192903

[3] Berkley, J. A. et al. Bacteremia among children admitted to a rural hospital in Kenya. *N. Engl. J. Med.* **352**, 39–47 (2005). 15635111 10.1056/NEJMoa040275

[4] Gilchrist, J. J. et al. BIRC6 modifies risk of invasive bacterial infection in Kenyan children. *Elife* **2022**, 1–24 (2022). 10.7554/eLife.77461 PMC9391038 35866869

[5] Bejon, P. et al. Defining childhood severe falciparum malaria for intervention studies. *PLoS Med.* **4**, 1333–1340 (2007). 10.1371/journal.pmed.0040251 PMC1949845 17713980

[6] Evans, J. A. et al. High mortality of infant bacteraemia clinically indistinguishable from severe malaria. *QJM Int. J. Med.* **97**, 591–597 (2004). 10.1093/qjmed/hch093 15317928

[7] Moyo, S. J. et al. Bacteraemia, malaria, and case fatality among children hospitalized with fever in Dar es Salaam, Tanzania. *Front. Microbiol.* **11**, 2118 (2020). 33013772 10.3389/fmicb.2020.02118 PMC7511546

[8] Sands, K. et al. Characterization of antimicrobial-resistant Gram-negative bacteria that cause neonatal sepsis in seven low- and middle-income countries. *Nat. Microbiol.* **6**, 512–523 (2021). 33782558 10.1038/s41564-021-00870-7 PMC8007471

[9] Carl, M. A. et al. Sepsis from the gut: the enteric habitat of bacteria that cause late-onset neonatal bloodstream infections. *Clin. Infect. Dis.* **58**, 1211–1218 (2014). 24647013 10.1093/cid/ciu084 PMC3982840

[10] Holmes, C. L., Anderson, M. T., Mobley, H. L. T. & Bachman, M. A. Pathogenesis of Gram-Negative bacteremia. *Clin. Microbiol. Rev*. **34**, e00234–20 (2021). 10.1128/CMR.00234-20 PMC8549824 33692149

[11] Schwartz, D. J. et al. Gut pathogen colonization precedes bloodstream infection in the neonatal intensive care unit. *Sci. Transl. Med*. **15**, eadg5562 (2023). 10.1126/scitranslmed.adg5562 PMC10259202 37134153

[12] Lee, C. C. et al. Gut dysbiosis, bacterial colonization and translocation, and neonatal sepsis in very-low-birth-weight preterm infants. *Front. Microbiol*. **12**, 746111 (2021). 10.3389/fmicb.2021.746111 PMC8529156 34690993

[13] Bischoff, S. C. et al. Intestinal permeability—a new target for disease prevention and therapy. *BMC Gastroenterol.* **14**, 1–25 (2014). 25407511 10.1186/s12876-014-0189-7 PMC4253991

[14] Dong, Y. & Speer, C. P. Late-onset neonatal sepsis: recent developments. *Arch. Dis. Child. Fetal Neonatal Ed.* **100**, F257–F263 (2015). 25425653 10.1136/archdischild-2014-306213 PMC4413803

[15] Sherman, M. P. New concepts of microbial translocation in the neonatal intestine: mechanisms and prevention. *Clin. Perinatol.* **37**, 565–579 (2010). 20813271 10.1016/j.clp.2010.05.006 PMC2933426

[16] Zaura, E. et al. Same exposure but two radically different responses to antibiotics: resilience of the salivary microbiome versus long-term microbial. *Am. Soc. Microbiol.* **6**, 1–11 (2015). 10.1128/mBio.01693-15 PMC4659469 26556275

[17] Nielsen, K. L. et al. Adaptation of *Escherichia coli* traversing from the faecal environment to the urinary tract. *Int. J. Med. Microbiol.* **306**, 595–603 (2016). 27825516 10.1016/j.ijmm.2016.10.005 PMC5209455

[18] Smith, A. et al. Concordance of gastrointestinal tract colonization and subsequent bloodstream infections with Gram-negative bacilli in very low birth weight infants in the neonatal intensive care unit. *Pediatr. Infect. Dis. J.* **29**, 831–835 (2010). 20539251 10.1097/INF.0b013e3181e7884f PMC2949271

[19] Chen, S. L. et al. Genomic diversity and fitness of *E. coli* strains recovered from the intestinal and urinary tracts of women with recurrent urinary tract infection. *Sci. Transl. Med.* **5**, 1252–1253 (2013). 10.1126/scitranslmed.3005497 PMC3695744 23658245

[20] Martin, R. M. et al. Molecular epidemiology of colonizing and infecting isolates of *Klebsiella pneumoniae*. *mSphere* **1**, 1–12 (2016). 10.1128/mSphere.00261-16 PMC5071533 27777984

[21] Bidet, P. et al. Comparative genomic analysis of ESBL-producing *Escherichia coli* from faecal carriage and febrile urinary tract infection in children: a prospective multicentre study. *JAC-Antimicrob. Resist.* **4**, 1–8 (2022). 10.1093/jacamr/dlac056 PMC9123598 35611261

[22] Kibwana, U. O. et al. Gastrointestinal colonization of extended-spectrum beta-lactamase-producing bacteria among children below five years of age hospitalized with fever in Dar es Salaam, Tanzania. *J. Glob. Antimicrob. Resist.* **30**, 107–114 (2022). 35667646 10.1016/j.jgar.2022.05.023

[23] Robins, D. et al. Genome sequence of antibiotic-resistant *Klebsiella quasipneumoniae* FSFC0558: a novel sequence type (ST8212). *Access Microbiol*. **7**, 000944.v3 (2025). 10.1099/acmi.0.000944.v3 PMC12032403 40292017

[24] Crawford, R. W. et al. Gallstones play a significant role in *Salmonella* spp. gallbladder colonization and carriage. *Proc. Natl Acad. Sci. USA* **107**, 4353–4358 (2010). 20176950 10.1073/pnas.1000862107 PMC2840110

[25] Gonzalez-Escobedo, G. & Gunn, J. S. Gallbladder epithelium as a niche for chronic *Salmonella* carriage. *Infect. Immun.* **81**, 2920–2930 (2013). 23732169 10.1128/IAI.00258-13 PMC3719562

[26] Dongol, S. et al. The microbiological and clinical characteristics of invasive *Salmonella* in gallbladders from cholecystectomy patients in Kathmandu, Nepal. *PLoS ONE* **7**, e47342 (2012). 10.1371/journal.pone.0047342 PMC3471863 23077595

[27] Worley, M. J. *Salmonella* Bloodstream Infections. *Trop. Med. Infect. Dis*. **8**, (2023). 10.3390/tropicalmed8110487 PMC10675298 37999606

[28] Parry, C. M., Hien, T. T., Dougan, G., White, N. J. & Farrar, J. J. Typhoid Fever. *N. Engl. J. Med.* **347**, 1770–1782 (2002). 12456854 10.1056/NEJMra020201

[29] Siu, L. K., Yeh, K. M., Lin, J. C., Fung, C. P. & Chang, F. Y. *Klebsiella pneumoniae* liver abscess: a new invasive syndrome. *Lancet Infect. Dis.* **12**, 881–887 (2012). 23099082 10.1016/S1473-3099(12)70205-0

[30] Nielsen, K. L. et al. Whole-genome comparison of urinary pathogenic *Escherichia coli* and faecal isolates of UTI patients and healthy controls. *Int. J. Med. Microbiol.* **307**, 497–507 (2017). 29031453 10.1016/j.ijmm.2017.09.007 PMC5792705

[31] Mora-Rillo, M. et al. Impact of virulence genes on sepsis severity and survival in *Escherichia coli* bacteremia. *Virulence* **6**, 93–100 (2015). 25654604 10.4161/21505594.2014.991234 PMC4603433

[32] Rodriguez-R, L. M. et al. An ANI gap within bacterial species that advances the definitions of intra-species units. *MBio* **15**, 1–14 (2024). 10.1128/mbio.02696-23 PMC10790751 38085031

[33] Watt, A. E. et al. Parameters for one health genomic surveillance of *Escherichia coli* from Australia. *Nat. Commun.* **16**, 1–14 (2025). 39747833 10.1038/s41467-024-55103-2 PMC11696363

[34] Gorrie, C. L. et al. Gastrointestinal carriage is a major reservoir of *Klebsiella pneumoniae* infection in intensive care patients. *Clin. Infect. Dis.* **65**, 208–215 (2017). 28369261 10.1093/cid/cix270 PMC5850561

[35] Ludden, C. et al. Defining nosocomial transmission of *Escherichia coli* and antimicrobial resistance genes: a genomic surveillance study. *Lancet Microbe* **2**, e472–e480 (2021). 34485958 10.1016/S2666-5247(21)00117-8 PMC8410606

[36] Geurtsen, J. et al. Genomics and pathotypes of the many faces of *Escherichia coli*. *FEMS Microbiol. Rev.* **2022**, 1–30 (2022). 10.1093/femsre/fuac031 PMC9629502 35749579

[37] Fabich, A. J. et al. Comparison of carbon nutrition for pathogenic and commensal *Escherichia coli* strains in the mouse intestine. *Infect. Immun.* **76**, 1143–1152 (2008). 18180286 10.1128/IAI.01386-07 PMC2258830

[38] Beaber, J. W., Hochhut, B. & Waldor, M. K. SOS response promotes horizontal dissemination of antibiotic resistance genes. *Nature* **427**, 72–74 (2004). 14688795 10.1038/nature02241

[39] Hastings, P., Rosenberg, S. & Slack, A. Antibiotic-induced lateral transfer of antibiotic resistance. *Trends Microbiol.* **12**, 401–404 (2004). 15337159 10.1016/j.tim.2004.07.003

[40] Nielsen, T. K., Browne, P. D. & Hansen, L. H. Antibiotic resistance genes are differentially mobilized according to resistance mechanism. *Gigascience* **11**, 1–17 (2022). 10.1093/gigascience/giac072 PMC9338424 35906888

[41] Davies, E. V., Winstanley, C., Fothergill, J. L. & James, C. E. The role of temperate bacteriophages in bacterial infection. *FEMS Microbiol. Lett.* **363**, fnw015 (2016). 26825679 10.1093/femsle/fnw015

[42] Wagner, P. L. & Waldor, M. K. Bacteriophage control of bacterial virulence. *Infect. Immun.* **70**, 3985–3993 (2002). 12117903 10.1128/IAI.70.8.3985-3993.2002 PMC128183

[43] Tinsley, C. R., Bille, E. & Nassif, X. Bacteriophages and pathogenicity: more than just providing a toxin? *Microbes Infect.* **8**, 1365–1371 (2006). 16698301 10.1016/j.micinf.2005.12.013

[44] Bottery, M. J. Ecological dynamics of plasmid transfer and persistence in microbial communities. *Curr. Opin. Microbiol.* **68**, 102152 (2022). 35504055 10.1016/j.mib.2022.102152 PMC9586876

[45] Thomas, C. M. & Nielsen, K. M. Mechanisms of, and barriers to, horizontal gene transfer between bacteria. *Nat. Rev. Microbiol.* **3**, 711–721 (2005). 16138099 10.1038/nrmicro1234

[46] Roberts, A. P., Mullany, P. & Wilson, M. Gene transfer in bacterial biofilms. *Methods Enzymol.* **336**, 60–65 (2001). 11398419 10.1016/s0076-6879(01)36578-3

[47] Alderliesten, J. B. et al. Effect of donor-recipient relatedness on the plasmid conjugation frequency: a meta-analysis. *BMC Microbiol* **20**, 1–10 (2020). 32456625 10.1186/s12866-020-01825-4 PMC7249681

[48] Hunter, P. R., Wilkinson, D. C., Catling, L. A. & Barker, G. C. Meta-analysis of experimental data concerning antimicrobial resistance gene transfer rates during conjugation. *Appl. Environ. Microbiol.* **74**, 6085–6090 (2008). 18708517 10.1128/AEM.01036-08 PMC2565951

[49] Kronstad, J. W. & Caza, M. Shared and distinct mechanisms of iron acquisition by bacterial and fungal pathogens of humans. *Front. Cell. Infect. Microbiol.* **4**, 1–23 (2013). 10.3389/fcimb.2013.00080 PMC3832793 24312900

[50] De Coster, W., D’Hert, S., Schultz, D. T., Cruts, M. & Van Broeckhoven, C. NanoPack: visualizing and processing long-read sequencing data. *Bioinformatics* **34**, 2666–2669 (2018). 29547981 10.1093/bioinformatics/bty149 PMC6061794

[51] Bouras, G. et al. Hybracter: enabling scalable, automated, complete and accurate bacterial genome assemblies. *Microb. Genom.* **10**, 1–15 (2024). 10.1099/mgen.0.001244 PMC11165638 38717808

[52] Wick, R. R., Judd, L. M., Gorrie, C. L. & Holt, K. E. Completing bacterial genome assemblies with multiplex MinION sequencing. *Microb. Genom.* **3**, 1–7 (2017). 10.1099/mgen.0.000132 PMC5695209 29177090

[53] Kolmogorov, M., Yuan, J., Lin, Y. & Pevzner, P. A. Assembly of long, error-prone reads using repeat graphs. *Nat. Biotechnol.* **37**, 540–546 (2019). 30936562 10.1038/s41587-019-0072-8

[54] Bouras, G., Sheppard, A. E., Mallawaarachchi, V. & Vreugde, S. Plassembler: an automated bacterial plasmid assembly tool. *Bioinformatics* **39**, 0–5 (2023). 10.1093/bioinformatics/btad409 PMC10326302 37369026

[55] Wick, R. R. & Holt, K. E. Polypolish: short-read polishing of long-read bacterial genome assemblies. *PLoS Comput. Biol.* **18**, 1–13 (2022). 10.1371/journal.pcbi.1009802 PMC8812927 35073327

[56] Shen, W., Le, S., Li, Y. & Hu, F. SeqKit: a cross-platform and ultrafast toolkit for FASTA/Q file manipulation. *PLoS ONE* **11**, 1–10 (2016). 10.1371/journal.pone.0163962 PMC5051824 27706213

[57] Wick, R. R., Schultz, M. B., Zobel, J. & Holt, K. E. Bandage: interactive visualization of de novo genome assemblies: Fig. 1. *Bioinformatics* **31**, 3350–3352 (2015). 26099265 10.1093/bioinformatics/btv383 PMC4595904

[58] Aziz, R. K. et al. The RAST server: rapid annotations using subsystems technology. *BMC Genom.* **9**, 75 (2008). 10.1186/1471-2164-9-75 PMC2265698 18261238

[59] Bortolaia, V. et al. ResFinder 4.0 for predictions of phenotypes from genotypes. *J. Antimicrob. Chemother.* **75**, 3491–3500 (2020). 32780112 10.1093/jac/dkaa345 PMC7662176

[60] Chen, L., Zheng, D., Liu, B., Yang, J. & Jin, Q. VFDB 2016: hierarchical and refined dataset for big data analysis - 10 years on. *Nucleic Acids Res.* **44**, D694–D697 (2016). 26578559 10.1093/nar/gkv1239 PMC4702877

[61] Doster, E. et al. MEGARes 2.0: a database for classification of antimicrobial drug, biocide and metal resistance determinants in metagenomic sequence data. *Nucleic Acids Res.* **48**, D561–D569 (2020). 31722416 10.1093/nar/gkz1010 PMC7145535

[62] Richter, M., Rosselló-Móra, R., Oliver Glöckner, F. & Peplies, J. JSpeciesWS: a web server for prokaryotic species circumscription based on pairwise genome comparison. *Bioinformatics* **32**, 929–931 (2016). 26576653 10.1093/bioinformatics/btv681 PMC5939971

[63] Argimón, S. et al. Rapid genomic characterization and global surveillance of *Klebsiella* using Pathogenwatch. *Clin. Infect. Dis.* **73**, S325–S335 (2021). 34850838 10.1093/cid/ciab784 PMC8634497

[64] Lam, M. M. C., Wick, R. R., Judd, L. M., Holt, K. E. & Wyres, K. L. Kaptive 2.0: updated capsule and lipopolysaccharide locus typing for the *Klebsiella pneumoniae* species complex. *Microb. Genom.* **8**, 1–12 (2022). 10.1099/mgen.0.000800 PMC9176290 35311639

[65] Brisse, S. et al. Wzi gene sequencing, a rapid method for determination of capsulartype for *Klebsiella* strains. *J. Clin. Microbiol.* **51**, 4073–4078 (2013). 24088853 10.1128/JCM.01924-13 PMC3838100

[66] Beghain, J. et al. ClermonTyping: an easy-to-use and accurate in silico method for *Escherichia* genus strain phylotyping. *Microb. Genom.* **4**, 1–8 (2018). 10.1099/mgen.0.000192 PMC6113867 29916797

[67] Clermont, O., Christenson, J. K., Denamur, E. & Gordon, D. M. The Clermont *Escherichia coli* phylo-typing method revisited: improvement of specificity and detection of new phylo-groups. *Environ. Microbiol. Rep.* **5**, 58–65 (2013). 23757131 10.1111/1758-2229.12019

[68] Letunic, I. & Bork, P. Interactive Tree of Life (iTOL) v6: recent updates to the phylogenetic tree display and annotation tool. *Nucleic Acids Res*. 78–82, 10.1093/nar/gkae268 (2024). 10.1093/nar/gkae268 PMC11223838 38613393

[69] Deatherage, D. E. & Barrick, J. E. Identification of mutations in laboratory-evolved microbes from next-generation sequencing data using breseq. In *Methods in Molecular Biology* Vol. 1151 (eds. Sun, L. & Shou, W.) 165–188 (Springer New York, 2014). 10.1007/978-1-4939-0554-6_12 PMC4239701 24838886

[70] Galperin, M. Y., Makarova, K. S., Wolf, Y. I. & Koonin, E. V. Expanded Microbial genome coverage and improved protein family annotation in the COG database. *Nucleic Acids Res.* **43**, D261–D269 (2015). 25428365 10.1093/nar/gku1223 PMC4383993

[71] Huerta-Cepas, J. et al. EggNOG 5.0: a hierarchical, functionally and phylogenetically annotated orthology resource based on 5090 organisms and 2502 viruses. *Nucleic Acids Res.* **47**, D309–D314 (2019). 30418610 10.1093/nar/gky1085 PMC6324079

[72] Camacho, C. et al. BLAST+: architecture and applications. *BMC Bioinform.* **10**, 421 (2009). 10.1186/1471-2105-10-421 PMC2803857 20003500

[73] Sim, N. L. et al. SIFT web server: predicting effects of amino acid substitutions on proteins. *Nucleic Acids Res*. **40**, 452–457 (2012). 10.1093/nar/gks539 PMC3394338 22689647

[74] Musicha, P. et al. Genomic landscape of extended-spectrum β-lactamase resistance in *Escherichia coli* from an urban African setting. *J. Antimicrob. Chemother.* **72**, 1602–1609 (2017). 28333330 10.1093/jac/dkx058 PMC5437524

[75] Musicha, P. et al. Genomic analysis of *Klebsiella pneumoniae* isolates from Malawi reveals acquisition of multiple ESBL determinants across diverse lineages. *J. Antimicrob. Chemother.* **74**, 1223–1232 (2019). 30778540 10.1093/jac/dkz032 PMC6477993

[76] Lewis, J. M. et al. Colonization dynamics of extended-spectrum beta-lactamase-producing Enterobacterales in the gut of Malawian adults. *Nat. Microbiol*. **7**, 1593–1604 (2022). 10.1038/s41564-022-01216-7 PMC9519460 36065064

[77] Graf, F. E. et al. Molecular mechanisms of re-emerging chloramphenicol susceptibility in extended-spectrum beta-lactamase-producing Enterobacterales. *Nat. Commun.* **15**, 9019 (2024). 39424629 10.1038/s41467-024-53391-2 PMC11489765

[78] Gilchrist, C. L. M. & Chooi, Y.-H. Clinker & clustermap.js: automatic generation of gene cluster comparison figures. *Bioinformatics* 1–3, 10.1093/bioinformatics/btab007 (2021). 10.1093/bioinformatics/btab007 33459763

[79] Goodman, R. rngoodman/blood-faecal-genomic-comparison. *Zenodo*10.5281/zenodo.17161337 (2025).

