# Supplementary material for: Comparative genomics of blood and faecal E. coli and K. pneumoniae isolates from neonates with bloodstream infections in Tanzania
Source: Commun Biol. 2025 Nov 18;8:1603. doi: 10.1038/s42003-025-09008-5 (PMC12627497; doi:10.1038/s42003-025-09008-5)
Supplement: Supplementary file 1 — Supplementary Information [file 42003_2025_9008_MOESM1_ESM.pdf]

Supplementary information

Comparative genomics of blood and faecal *E. coli* and *K. pneumoniae* isolates from neonates with blood stream infections in Tanzania

Richard N. Goodman[1], Sabrina J. Moyo[1,2], Ilinca Memelis[1], Aakash Khanijau[1], Joel Manyahi[3], Upendo O. Kibwana [3], Said Aboud [3,4], Bjørn Blomberg[2,5], Nina Langeland[2,5,6], Adam P. Roberts[1*]

1. Department of Tropical Disease Biology, Liverpool School of Tropical Medicine, Pembroke Place, Liverpool, L3 5QA, UK
2. Department of Clinical Science, University of Bergen, Bergen, Norway
3. Department of Microbiology and Immunology, Muhimbili University of Health and Allied Sciences, Dar es Salaam, Tanzania
4. National Institute of Medical Research (NIMR), Dar es Salaam, Tanzania
5. Department of Medicine, Haukeland University Hospital, Bergen, Norway
6. Norwegian Institute of Public Health, Oslo, Norway

*Corresponding author

Adam P. Roberts. Department of Tropical Disease Biology, Liverpool School of Tropical Medicine, Pembroke Place, Liverpool, L3 5QA, UK.

Adam.Roberts@lstmed.ac.uk

Sample	Assembly method	file format	type	number of seqs	sum length	min length	average length	max length	Q1	Q2	Q3	N50	GC(%)
FSBL0558	hybracter	FASTA	DNA	3	5,544,499	4,259	1,848,166.30	5,390,483	77,008	149,757	2,770,120	5,390,483	57.13
FSBL1386	hybracter	FASTA	DNA	2	5,484,582	143,420	2,742,291	5,341,162	143,420	2,742,291	5,341,162	5,341,162	57.33
FSBL1448	flye	FASTA	DNA	6	5,785,608	5,763	964,268	5,328,540	5,913	112,375	220,642	5,328,540	56.71
FSBL1654	hybracter	FASTA	DNA	28	5,065,408	106	180,907.40	4,961,302	125	291.5	1,533.50	4,961,302	50.65
FSBL1925	hybracter	FASTA	DNA	6	5,479,388	3,676	913,231.30	5,242,001	4,907	19,816.50	189,171	5,242,001	57.31
FSBL2071	hybracter	FASTA	DNA	4	5,296,687	1,378	1,324,171.80	5,052,976	49,500	121,166.50	2,598,843.50	5,052,976	50.72
FSBL2111	hybracter	FASTA	DNA	4	5,609,861	3,574	1,402,465.30	5,488,634	3,794	58,826.50	2,801,136.50	5,488,634	57.19
FSBL2112	hybracter	FASTA	DNA	4	5,609,862	3,574	1,402,465.50	5,488,634	3,794	58,827	2,801,137	5,488,634	57.19
FSBL2130	hybracter	FASTA	DNA	8	5,895,662	3,301	736,957.80	5,278,286	3,392.50	42,972.50	260,672.50	5,278,286	56.7
FSBL2155	hybracter	FASTA	DNA	4	5,914,917	100,163	1,478,729.30	5,324,353	146,693	245,200.50	2,810,765.50	5,324,353	56.68
FSBL2240	hybracter	FASTA	DNA	4	5,108,105	2,125	1,277,026.30	5,009,705	3,099.50	48,137.50	2,550,953	5,009,705	50.65
FSBL2258	hybracter	FASTA	DNA	5	5,129,516	2,125	1,025,903.20	4,939,041	5,164	90,985	92,201	4,939,041	50.62
FSBL2265	hybracter	FASTA	DNA	9	5,997,171	3,301	666,352.30	5,278,317	3,468	75,683	226,098	5,278,317	56.66
FSFC0558	hybracter	FASTA	DNA	2	5,269,471	166,709	2,634,735.50	5,102,762	166,709	2,634,735.50	5,102,762	5,102,762	58.02
FSFC1386	hybracter	FASTA	DNA	3	5,490,501	6,415	1,830,167	5,340,663	74,919	143,423	2,742,043	5,340,663	57.31
FSFC1448	flye	FASTA	DNA	5	5,786,169	10,122	1,157,233.80	5,328,480	14,596	212,329	220,642	5,328,480	56.71
FSFC1654	unicycler	FASTA	DNA	28	5,042,889	131	180,103.20	1,190,061	843.5	9,908.50	172,451	933,194	50.64
FSFC1925	hybracter	FASTA	DNA	4	5,684,240	63,275	1,421,060	5,290,046	87,407	165,459.50	2,754,713	5,290,046	57.03
FSFC2071	hybracter	FASTA	DNA	3	5,295,994	98,286	1,765,331.30	5,052,997	121,498.50	144,711	2,598,854	5,052,997	50.72
FSFC2111K	hybracter	FASTA	DNA	2	5,593,489	229,590	2,796,744.50	5,363,899	229,590	2,796,744.50	5,363,899	5,363,899	57.07
FSFC2112	hybracter	FASTA	DNA	4	5,608,700	3,574	1,402,175	5,488,573	3,794	58,276.50	2,800,556	5,488,573	57.19
FSFC2130	hybracter	FASTA	DNA	9	5,885,363	873	653,929.20	5,278,286	3,301	4,761	224,982	5,278,286	56.71
FSFC2155	hybracter	FASTA	DNA	3	5,537,134	244,643	1,845,711.30	4,924,353	306,390.50	368,138	2,646,245.50	4,924,353	57.17
FSFC2240	unicycler	FASTA	DNA	27	5,084,135	131	188,301.30	2,839,153	328	1,225	11,260	2,839,153	50.64
FSFC2258	hybracter	FASTA	DNA	5	5,129,448	2,125	1,025,889.60	4,939,014	5,164	90,944	92,201	4,939,014	50.62
FSFC2265	hybracter	FASTA	DNA	27	5,645,840	232	209,105.20	5,172,443	1,970.50	4,761	22,757.50	5,172,443	57

Supplementary Table 1: QC of assembled genomes used in analysis. The reads were assembled with either hybracter (*n*=22), unicycler (*n*=2) or flye (*n*=2).

Isolate	Species	Sample source:	ID number	Hospital	Year	Age on admission	Duration	Antibiotics before admission	Antibiotics before culture	Antibiotics
FSFC0558	*K. quasipneumoniae similipneumoniae*	Faecal	FS0558	MNH	2017	1 day (<24hrs)	(<24hrs)	No	Yes	Amoxycillin+genta
FSFC1386	*K. pneumoniae*	Faecal	FS1386	Temeke	2018	2 days	2	No	Yes	Amoxycillin+genta
FSFC1448	*K. pneumoniae*	Faecal	FS1448	Temeke	2018	2 days	2	No	Yes	Amoxycillin+genta
FSFC1654	*E. coli*	Faecal	FS1654	Temeke	2018	1 day	1 day	No	Yes	Amoxycillin+genta
FSFC1925	*K. pneumoniae*	Faecal	FS1925	Amana	2018	3 days	2 days	No	Yes	Amoxycillin /then Ceftriaxone +genta
FSFC2071	*E. coli*	Faecal	FS2071	Temeke	2018	4 days	2 days	No	Yes	Amoxycillin+genta
FSFC2111K	*K. pneumoniae*	Faecal	FS2111	Amana	2018	2 days	1 day	No	Yes	Amoxycillin /then Ceftriaxone +genta
FSFC2112	*K. pneumoniae*	Faecal	FS2112	Amana	2018	3 days	2 days	No	Yes	Amoxycillin /then Ceftriaxone +genta
FSFC2130	*K. pneumoniae*	Faecal	FS2130	Amana	2018	3 days	2 days	No	Yes	Amoxycillin /then Ceftriaxone +genta
FSFC2155	*K. pneumoniae*	Faecal	FS2155	Amana	2018	1 day	1 day	No	Yes	Amoxycillin+genta
FSFC2240	*E. coli*	Faecal	FS2240	Temeke	2018	16 days	2 days	No	Yes	Amoxycillin+genta
FSFC2258	*E. coli*	Faecal	FS2258	Temeke	2018	1 day	1 day	No	Yes	Amoxycillin+genta
FSFC2265	*K. pneumoniae*	Faecal	FS2265	Temeke	2018	19 days	3 days	No	Yes	Amoxycillin+genta
FSBL0558	*K. pneumoniae*	Blood	FS0558	MNH	2018	1 day (<24hrs)	(<24hrs)	No	Yes	Amoxycillin+genta
FSBL1386	*K. pneumoniae*	Blood	FS1386	Temeke	2018	2 days	2	No	Yes	Amoxycillin+genta
FSBL1448	*K. pneumoniae*	Blood	FS1448	Temeke	2018	2 days	2	No	Yes	Amoxycillin+genta
FSBL1654	*E. coli*	Blood	FS1654	Temeke	2018	1 day	1 day	No	Yes	Amoxycillin+genta
FSBL1925	*K. pneumoniae*	Blood	FS1925	Amana	2018	3 days	2 days	No	Yes	Amoxycillin /then Ceftriaxone +genta
FSBL2071	*E. coli*	Blood	FS2071	Temeke	2018	4 days	2 days	No	Yes	Amoxycillin+genta
FSBL2111	*K. pneumoniae*	Blood	FS2111	Amana	2018	2 days	1 day	No	Yes	Amoxycillin /then Ceftriaxone +genta
FSBL2112	*K. pneumoniae*	Blood	FS2112	Amana	2018	3 days	2 days	No	Yes	Amoxycillin /then Ceftriaxone +genta
FSBL2130	*K. pneumoniae*	Blood	FS2130	Amana	2018	3 days	2 days	No	Yes	Amoxycillin /then Ceftriaxone +genta
FSBL2155	*K. pneumoniae*	Blood	FS2155	Amana	2018	1 day	1 day	No	Yes	Amoxycillin+genta
FSBL2240	*E. coli*	Blood	FS2240	Temeke	2018	16 days	2 days	No	Yes	Amoxycillin+genta
FSBL2258	*E. coli*	Blood	FS2258	Temeke	2018	1 day	1 day	No	Yes	Amoxycillin+genta
FSBL2265	*K. pneumoniae*	Blood	FS2265	Temeke	2018	19 days	3 days	No	Yes	Amoxycillin+genta

Supplementary Table 2: List of clinical metadata associated with each blood and faecal paired isolates. The ID number refers to individual patients.

Breseq SNP and INDELs	
Sample	Frequency
FS1386	1
FS1448	8
FS1654	10
FS2071	97
FS2112	4
FS2130	6
FS2258	23
FS2240	34
FS2155	516

Average	77.66
Min	1
Max	516
Median	10
Standard deviation	167.06

Supplementary Table 3: SNP and small INDEL (<100bp) frequencies from breseq whole genomic comparison.

Isolate name	Sample source	Isolate name	Contig name	length (bp)	GC (%)	Circular	Sample source	Isolate name	Contig name	length	GC	Circular	Difference in blood relative to fecal (+/-
FS1386	Fecal	FSFC1386	chromosome	5340663	57.48	FALSE	Blood	FSBL1386	chromosome	5341162	57.48	TRUE	499
			plasmid 1	143423	51.87	TRUE			plasmid 1	143420	51.87	TRUE	-3
			Plasmid 2	6415	40.12	TRUE							-6415
FS1448*		FSFC1448	chromosome	5328480	57.4	FALSE		FSBL1448	chromosome	5328540	57.4	FALSE	60
			plasmid 1	220642	52.08	FALSE			plasmid 1	220642	52.08	FALSE	0
			plasmid 2	212329	45.19	FALSE			plasmid 2	212332	45.19	FALSE	3
			plasmid 3	14596	43.45	FALSE			plasmid 3	12418	52.48	FALSE	-2178
			plasmid 4	10122	53.39	FALSE			plasmid 4	5913	43.62	FALSE	-4209
									plasmid 5	5763	42.11	FALSE	5763
FS2071		FSFC2071	Chromsome	5052997	50.72	TRUE		FSBL2071	chromosome	5052976	50.72	TRUE	-21
			plasmid 1	144711	52.6	TRUE			plasmid 1	144711	52.6	TRUE	0
			Plasmid 2	98286	47.91	TRUE			plasmid 2	97622	47.92	TRUE	-664
									plasmid 3	1378	47.39	FALSE	1378
FS2112		FSFC2112	chromosome	5488573	57.3	TRUE		FSBL2112	chromosome	5488634	57.3	TRUE	61
			plasmid 1	112539	52.78	TRUE			plasmid 1	113640	52.77	TRUE	1101
			plasmid 2	4014	43.72	TRUE			plasmid 2	4014	43.72	TRUE	0
			plasmid 3	3574	43.84	TRUE			plasmid 3	3574	43.84	TRUE	0
FS2130		FSFC2130	chromosome	5278286	57.51	TRUE		FSBL2130	chromosome	5278286	57.51	TRUE	0
			plasmid 1	287690	47.06	FALSE			plasmid 1	296347	47.17	TRUE	8657
			plasmid 2	224982	51.98	TRUE			plasmid 2	224998	51.98	TRUE	16
			plasmid 3	81179	54	TRUE			plasmid 3	81184	54.05	TRUE	5
			plasmid 4	4761	44.42	TRUE			plasmid 4	4761	44.42	TRUE	0
			plasmid 5	3317	46.28	TRUE			plasmid 5	3468	45.13	TRUE	151
			plasmid 6	3301	43.02	TRUE			plasmid 6	3317	46.28	TRUE	16
			plasmid 7	974	40.76	FALSE			plasmid 7	3301	43.02	TRUE	2327
			plasmid 8	873	43.64	FALSE							
FS2155		FSFC2155	chromosome	4924353	57.28	FALSE		FSBL2155	chromosome	5324353	57.42	TRUE	400000
			plasmid 1	368138	58.77	FALSE			plasmid 1	297178	47.19	TRUE	-70960
			plasmid 2	244643	52.58	FALSE			plasmid 2	193223	52.01	TRUE	-51420
									plasmid 3	100163	54.24	TRUE	100163
FS2258		FSFC2258	chromosome	4939014	50.62	TRUE		FSBL2258	chromosome	4939041	50.62	TRUE	27
			plasmid 1	92201	51.41	TRUE			plasmid 1	92201	51.41	TRUE	0
			plasmid 2	90944	50.12	TRUE			plasmid 2	90985	50.11	TRUE	41
			plasmid 3	5164	47.52	TRUE			plasmid 3	5164	47.52	TRUE	0
			plasmid 4	2125	47.11	TRUE			plasmid 4	2125	47.11	TRUE	0

Supplementary Table 4: Genome assembly statistics by contigs across highly related pairs with hybrid assemblies. Isolates from FS1654 and FS2240, although highly related pairs were omitted as their assemblies have > 25 contigs. Isolates from FS2155 are included for reference although not classed as a highly related pair. Difference is calculated by subtracting contig sizes of blood isolate by contig sizes of faecal isolate.

Sample	CFU ml^{-1}	CFU $100\mu l^{-1}$	Growth on LB+RIF (20µg/ml) after 16hrs				Mutation Frequency
			1	2	3	Average	
FS1386	1.74×10^{7}	1.74×10^{6}	0	0	0	0	0
FS1448	1.69×10^{7}	1.69×10^{6}	0	1	1	0.66666667	3.94×10^{-7}
FS2071	1.56×10^{7}	1.56×10^{6}	0	0	0	0	0
FS2112	1.74×10^{7}	1.74×10^{6}	0	1	2	1	5.75×10^{-7}
FS2130 (RIF^{R} ARR-3)	1.77×10^{7}	1.77×10^{6}	L	L	L	L	1
FS2155	1.25×10^{7}	1.25×10^{6}	2	0	0	0.66666667	5.33×10^{-7}
FS2258	1.42×10^{7}	1.42×10^{6}	0	0	0	0	0

Supplementary Table 5: Rifampicin mutation assay. Mutation frequency was calculated by dividing the number of rifampicin-resistant colonies by the total number of viable cells plated. LB agar plates were supplemented with rifampicin at a concentration of 20µg/ml. FS2130 carried the *arr-3* gene which confers resistance to rifampicin and therefore was observed as a lawn on the rifampicin supplemented LB agar plates. L, lawn; RIF, rifampicin; RIF^{R} rifampicin resistant.

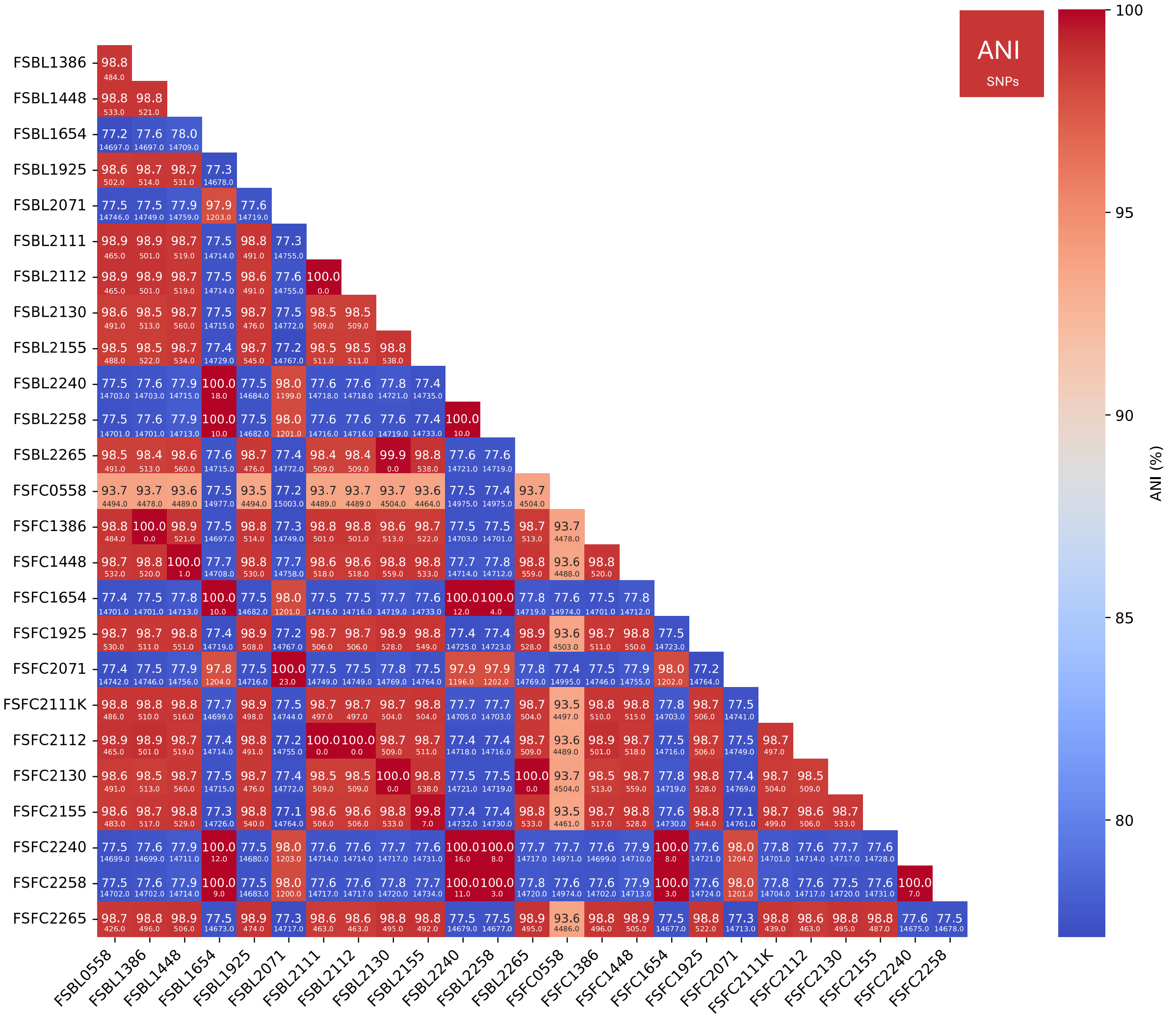

Supplementary Figure 1: Average nucleotide identities (ANI) and single nucleotide polymorphisms (SNPs) across all 13 paired isolates.

ANI was calculated using the BLAST alignment algorithm (ANIb). The core genome SNPs are displayed in each cell below the ANI %, this was calculated using snippy v4.6.3 and snp-dist using FSFC1386 as reference.

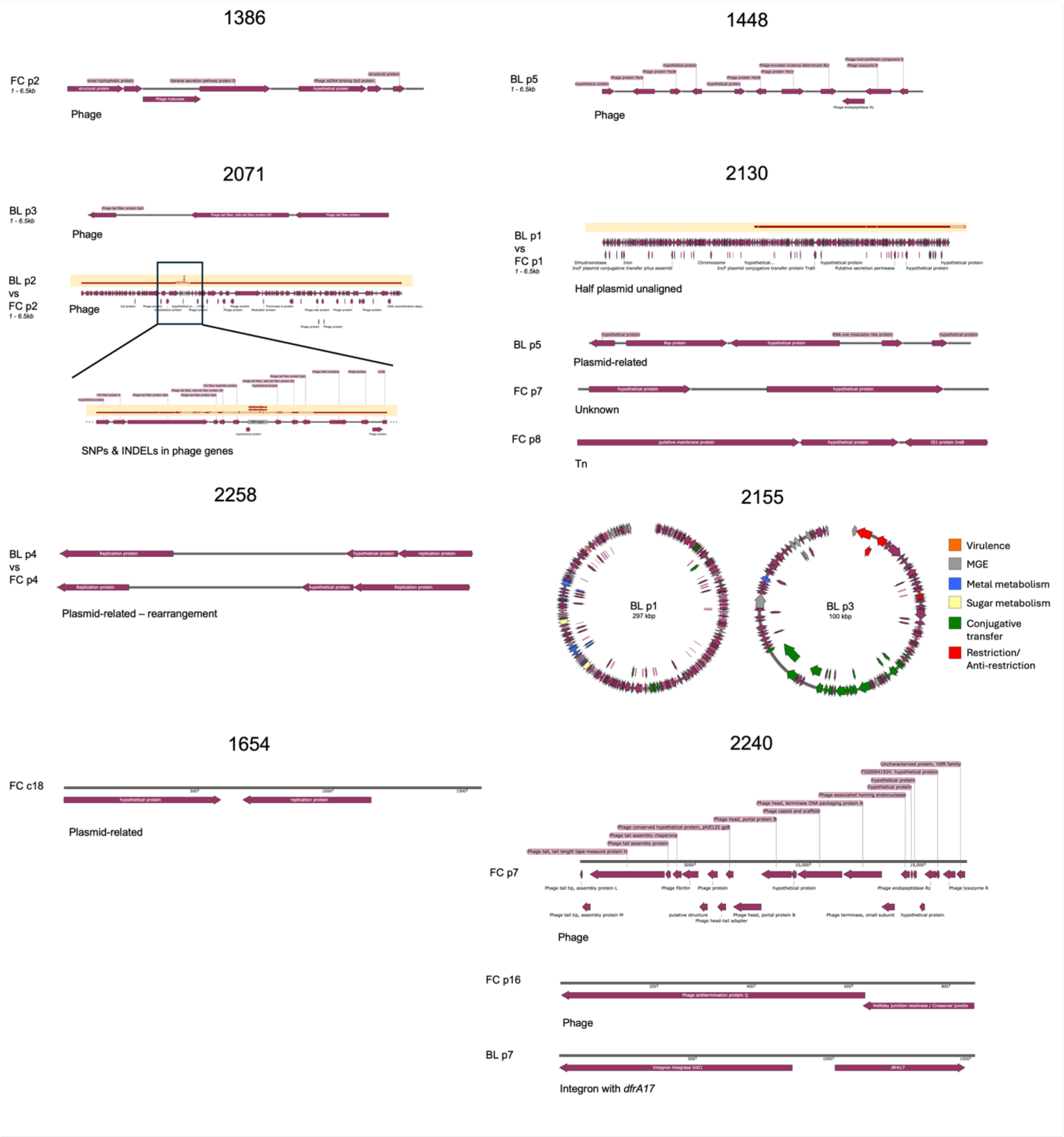

Supplementary Figure 2: Loss and gain of genetic regions between contigs of highly related pairs

Connections from Figure 4 were analysed and any contigs or regions of contigs not shared were visualised by Snapgene. In FSFC1386 there is a 6415bp contig (FC plasmid 2) that is not in FSBL1386, this contains mainly phage genes. FS1448 and FS2071 contain contigs with phage related genes in the blood isolate but not in the faecal isolate. Plasmid 7 and 8 of the faecal isolate FSFC2130 are not found in the blood

isolate FSBL2130, faecal plasmid 7 contains an IS*1* Tn, whereas faecal plasmid 8 contains only unknown genes. However, the blood isolate FSBL2130 contains a plasmid not found in the faecal isolate (BL p5) which harbours a *rep* protein and hypothetical proteins.

Over half (45.7%; 135,460bp/296,347bp) of the length of plasmids BL p1 and FC p1 of FS2071 do not align at 99.9% sequence identity, this means it is likely that there is loss of entire sections or large genomic movement such as recombination.

Between isolates of FS2258, blood plasmid 4 and faecal plasmid 4 do not align but this is due to a rearrangement in a replication protein.

The blood and faecal genomes of FS2155 have the largest disparities of all paired samples, with a 400kb disparity in chromosome size however this could be explained by the insertion of FC p1 into the chromosome. FSBL2155 p2 is mainly accounted for in FC p2, however the blood isolate has two extra plasmids BL p1 and BL p3. BL p1 is a 297kb plasmid containing IncF conjugative *tra* genes and the vap toxin producing gene. BL p1 shares the *fec* operon, encoding the Iron(III) dicitrate transport system, and the *lac* operon with FC p2. BL p3 is a 100kb plasmid with *tra* genes and a type I restriction-modification system, it shares an IS*26* with FC p2 seen as the connection on Figure 4 but no additional homology.

FS1654 and FS2240 were visualised differently based on the method of assembly for the faecal isolates (unicycler rather than hybracter) which is why the contigs are named different (e.g. c1 to c28 for FS1654), with some of these contigs being in the 100s of bp. With FS1654 there is one additional contig in the faecal c18 and 22 additional small contigs (p6 to p27) in the blood. However, this is likely a fragmented set of FC c18 as this contig aligns to these 15 small BL contigs at between 98.942% and 99.742%. FC

c18 is 1552bp in length and contains a *rep* gene and hypothetical protein gene, the 15 small blood contigs contain both genes or just the *rep* gene. There are 7 contigs from the blood isolate FSBL1654 which share no homology with the faecal isolate FSFC1654, one of these is BL p7 which contains a *dfrA17* gene, previously shown to be the one difference in AMR profiles across the highly related pairs (see Supplementary Table 3), the rest are small contigs with either hypothetical genes or no genes.

With FS2240 there are additional contigs in the faecal isolates: c16 and c7. However, c7 aligns to the BL chromosome with 99.875% identity across its length (16,812bp) and c16 aligns to the chromosome with 99.647% across its length (851bp) likely meaning these were misassembled parts of the chromosome. FC c7 contains 24 genes, all phage or pro-phage related including those involved in the assembly of the phage head and tail and FC c16 contains two genes a phage antitermination protein gene and a Holliday junction resolvase gene: *rusA*. It is well known that phage genes being repetitive in nature are difficult to assemble if the reads don’t span the length of the longest repeat.
