# Supplementary material for: Comparative genomics of blood and faecal E. coli and K. pneumoniae isolates from neonates with bloodstream infections in Tanzania
Source: Commun Biol. 2025 Nov 18;8:1603. doi: 10.1038/s42003-025-09008-5 (PMC12627497; doi:10.1038/s42003-025-09008-5)
Supplement: Supplementary file 2 — Description of Additional Supplementary Materials [file 42003_2025_9008_MOESM2_ESM.pdf]

Description of Additional Supplementary Files

File name: Supplementary Data 1

Description: Resfinder, Virulence factor and MEGARES database screening of 8 highly related pairs. All pairs were hybrid assembled with hybracter except FS1448 where a flye assembly of long reads is used and FS1654 and FS2240 where a unicycler hybrid assembly was used.

File name: Supplementary Data 2

Description: Breseq comparisons of FSFC2155 hybrid assembly against FSBL2155 long reads. 516 SNPs in 289 genes are displayed grouped by gene classification.
