# Supplementary material for: Comparative genomics of blood and faecal E. coli and K. pneumoniae isolates from neonates with bloodstream infections in Tanzania
Source: Commun Biol. 2025 Nov 18;8:1603. doi: 10.1038/s42003-025-09008-5 (PMC12627497; doi:10.1038/s42003-025-09008-5)
Supplement: Supplementary file 5 — Reporting Summary [file 42003_2025_9008_MOESM5_ESM.pdf]

Corresponding author(s): Adam P. Roberts

Last updated by author(s): Sep 24, 2025

Reporting Summary

Nature Portfolio wishes to improve the reproducibility of the work that we publish. This form provides structure for consistency and transparency in reporting. For further information on Nature Portfolio policies, see our Editorial Policies and the Editorial Policy Checklist.

Statistics

For all statistical analyses, confirm that the following items are present in the figure legend, table legend, main text, or Methods section.

n/a	Confirmed	
☐	☒	The exact sample size (*n*) for each experimental group/condition, given as a discrete number and unit of measurement
☐	☒	A statement on whether measurements were taken from distinct samples or whether the same sample was measured repeatedly
☒	☐	The statistical test(s) used AND whether they are one- or two-sided<br>*Only common tests should be described solely by name; describe more complex techniques in the Methods section.*
☒	☐	A description of all covariates tested
☒	☐	A description of any assumptions or corrections, such as tests of normality and adjustment for multiple comparisons
☒	☐	A full description of the statistical parameters including central tendency (e.g. means) or other basic estimates (e.g. regression coefficient) AND variation (e.g. standard deviation) or associated estimates of uncertainty (e.g. confidence intervals)
☒	☐	For null hypothesis testing, the test statistic (e.g. *F*, *t*, *r*) with confidence intervals, effect sizes, degrees of freedom and *P* value noted<br>*Give P values as exact values whenever suitable.*
☒	☐	For Bayesian analysis, information on the choice of priors and Markov chain Monte Carlo settings
☒	☐	For hierarchical and complex designs, identification of the appropriate level for tests and full reporting of outcomes
☒	☐	Estimates of effect sizes (e.g. Cohen's *d*, Pearson's *r*), indicating how they were calculated

Our web collection on statistics for biologists contains articles on many of the points above.

Software and code

Policy information about availability of computer code

Data collection	Short and long read sequencing was carried out on paired blood and faecal isolates. Enrolment of participants, collection of blood samples and bacterial isolation was carried out as described previously (Moyo, S. J. et al. Bacteraemia, Malaria, and Case Fatality Among Children Hospitalized With Fever in Dar es Salaam, Tanzania. Front. Microbiol. 11, 2118 (2020)). The faecal samples were collected as rectal swabs from each patient and bacteria was isolated as previously described (Kibwana, U. O. et al. Gastrointestinal colonization of extended-spectrum beta-lactamase-producing bacteria among children below five years of age hospitalized with fever in Dar es Salaam, Tanzania. J. Glob. Antimicrob. Resist. 30, 107–114 (2022).)
Data analysis	MinKNOW v22.08.9, guppy v6.4.2, Nanoplot v1.38.1, hybracter v0.6.0, unicycler v0.4.8, flye v2.9.2, Bandage v0.8.1, abricate v1.0.1, JSpeciesWS v4.1.1, snippy v4.6.3, snp-dists v0.8.2, pandas v 2.2.3, matplotlib v 3.9.4, seaborn v 0.13.2 , numpy v 2.2.0, gubbins v2.3.4, snp-sites v2.5.1, IQ-tree v1.6.1, Interactive Tree Of Life (iTOL) v6, dplyr v1.1.4, ggplot v3.5.1, Breseq v0.38.3, eggNOG v5.0, pheatmap v1.0.12, circlize v0.4.16, clinker v0.0.31.<br><br>The code and data to replicate the analysis is available at https://rngoodman.github.io/blood-faecal-genomic-comparison and on a mirrored Zenodo repository: https://doi.org/10.5281/zenodo.1716133679.

For manuscripts utilizing custom algorithms or software that are central to the research but not yet described in published literature, software must be made available to editors and reviewers. We strongly encourage code deposition in a community repository (e.g. GitHub). See the Nature Portfolio guidelines for submitting code & software for further information.

Data

Policy information about availability of data

All manuscripts must include a data availability statement. This statement should provide the following information, where applicable:

- Accession codes, unique identifiers, or web links for publicly available datasets
- A description of any restrictions on data availability
- For clinical datasets or third party data, please ensure that the statement adheres to our policy

Reads from all isolates sequenced as part of this study have been submitted to the Sequence Read Archive (SRA) of the National Centre for Biotechnology Information (NCBI) under the project ID PRJNA1254181. Supplementary figures 1-2, supplementary tables 1-5 and supplementary data 1-2 are accessible in the supplementary material of this manuscript.

Research involving human participants, their data, or biological material

Policy information about studies with human participants or human data. See also policy information about sex, gender (identity/presentation), and sexual orientation and race, ethnicity and racism.

Reporting on sex and gender	Sex and gender was not reported in this study
Reporting on race, ethnicity, or other socially relevant groupings	Race and ethnicity was not reported in this study
Population characteristics	The samples were collected from patients under the age of 19 days with fevers lasting 1 – 3 days in three hospitals in Dar es Salaam, Tanzania. Supplementary Table 2 contains more details of the characteristics of the human research participants.
Recruitment	Enrolment of participants is described in a previous publication (Moyo, S. J. et al. Bacteraemia, Malaria, and Case Fatality Among Children Hospitalized With Fever in Dar es Salaam, Tanzania. Front. Microbiol. 11, 2118 (2020)).
Ethics oversight	The original sample collection was part of a study approved by the Senate Research and Publications Committee of Muhimbili University of Health and Allied Sciences, National Institute of Medical Research, Tanzania and the Regional Committee for Medical and Health Research Ethics (REK), Norway.

Note that full information on the approval of the study protocol must also be provided in the manuscript.

Field-specific reporting

Please select the one below that is the best fit for your research. If you are not sure, read the appropriate sections before making your selection.

☒ Life sciences ☐ Behavioural & social sciences ☐ Ecological, evolutionary & environmental sciences

For a reference copy of the document with all sections, see nature.com/documents/nr-reporting-summary-flat.pdf

Life sciences study design

All studies must disclose on these points even when the disclosure is negative.

Sample size	The study described in this manuscript analysed 13 blood and faecal isolate pairs (n = 26).
Data exclusions	No data were excluded from the analysis. However not all the originally paired isolates could be found and sequenced, therefore only 26 out of the original 32 isolates could be sequenced as pairs. By not sequencing these we may have lost information about genes or SNPs involved in bacterial translocation to the blood and we cannot predict how this may have biased the results.
Replication	Experimental replication is not applicable to observational studies.
Randomization	This is a non-randomised study, this is not applicable to observational studies.
Blinding	This is a nonblinded study. Blinding is not relevant to observational studies such as this.

Reporting for specific materials, systems and methods

We require information from authors about some types of materials, experimental systems and methods used in many studies. Here, indicate whether each material, system or method listed is relevant to your study. If you are not sure if a list item applies to your research, read the appropriate section before selecting a response.

Materials & experimental systems

n/a	Involved in the study
☒	☐ Antibodies
☒	☐ Eukaryotic cell lines
☒	☐ Palaeontology and archaeology
☒	☐ Animals and other organisms
☒	☐ Clinical data
☒	☐ Dual use research of concern
☒	☐ Plants

Methods

n/a	Involved in the study
☒	☐ ChIP-seq
☒	☐ Flow cytometry
☒	☐ MRI-based neuroimaging

Plants

Seed stocks

Report on the source of all seed stocks or other plant material used. If applicable, state the seed stock centre and catalogue number. If plant specimens were collected from the field, describe the collection location, date and sampling procedures.

Novel plant genotypes

Describe the methods by which all novel plant genotypes were produced. This includes those generated by transgenic approaches, gene editing, chemical/radiation-based mutagenesis and hybridization. For transgenic lines, describe the transformation method, the number of independent lines analyzed and the generation upon which experiments were performed. For gene-edited lines, describe the editor used, the endogenous sequence targeted for editing, the targeting guide RNA sequence (if applicable) and how the editor was applied.

Authentication

Describe any authentication procedures for each seed stock used or novel genotype generated. Describe any experiments used to assess the effect of a mutation and, where applicable, how potential secondary effects (e.g. second site T-DNA insertions, mosiacism, off-target gene editing) were examined.
